# Supplementary material for: Testing of the Survivin Suppressant YM155 in a Large Panel of Drug-Resistant Neuroblastoma Cell Lines
Source: Cancers (Basel). 2020 Mar 2;12(3):577. doi: 10.3390/cancers12030577 (PMC7139505; doi:10.3390/cancers12030577)
Supplement: Supplementary file 1 [file cancers-12-00577-s001.zip › Michaelis et al_Supplements/Michaelis et al_Figure 5_revised.pptx]

## Slide 1
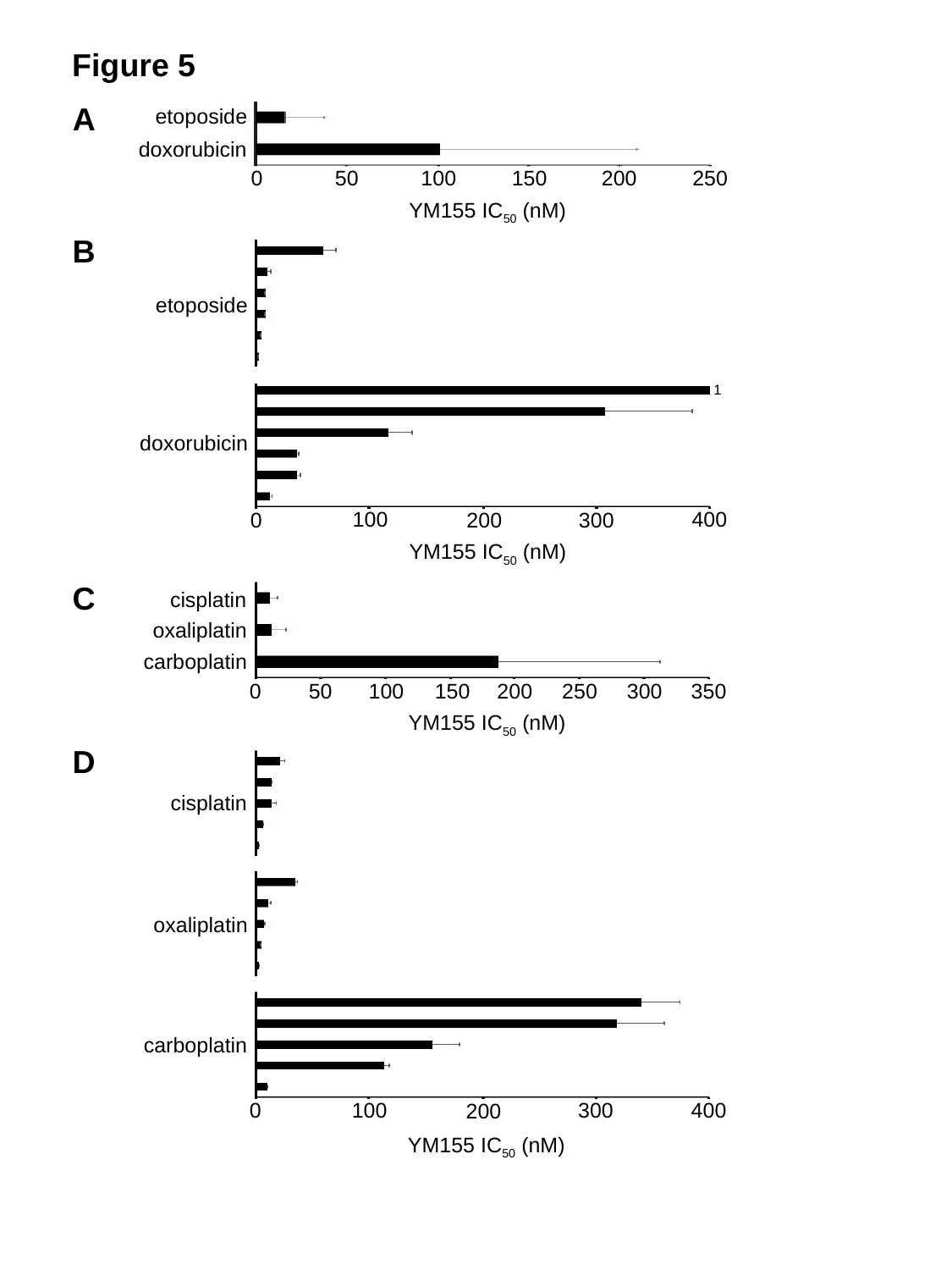

Figure 5
A
etoposide
doxorubicin
100
250
0
150
50
200
YM155 IC50 (nM)
B
etoposide
1
doxorubicin
100
400
0
300
200
YM155 IC50 (nM)
C
cisplatin
oxaliplatin
carboplatin
100
350
0
300
50
150
250
200
YM155 IC50 (nM)
D
cisplatin
oxaliplatin
carboplatin
100
400
0
300
200
YM155 IC50 (nM)
