# Supplementary material for: Testing of the Survivin Suppressant YM155 in a Large Panel of Drug-Resistant Neuroblastoma Cell Lines
Source: Cancers (Basel). 2020 Mar 2;12(3):577. doi: 10.3390/cancers12030577 (PMC7139505; doi:10.3390/cancers12030577)
Supplement: Supplementary file 1 [file cancers-12-00577-s001.zip › Michaelis et al_Supplements/Michaelis et al_Figure 2_revised.pptx]

## Slide 1
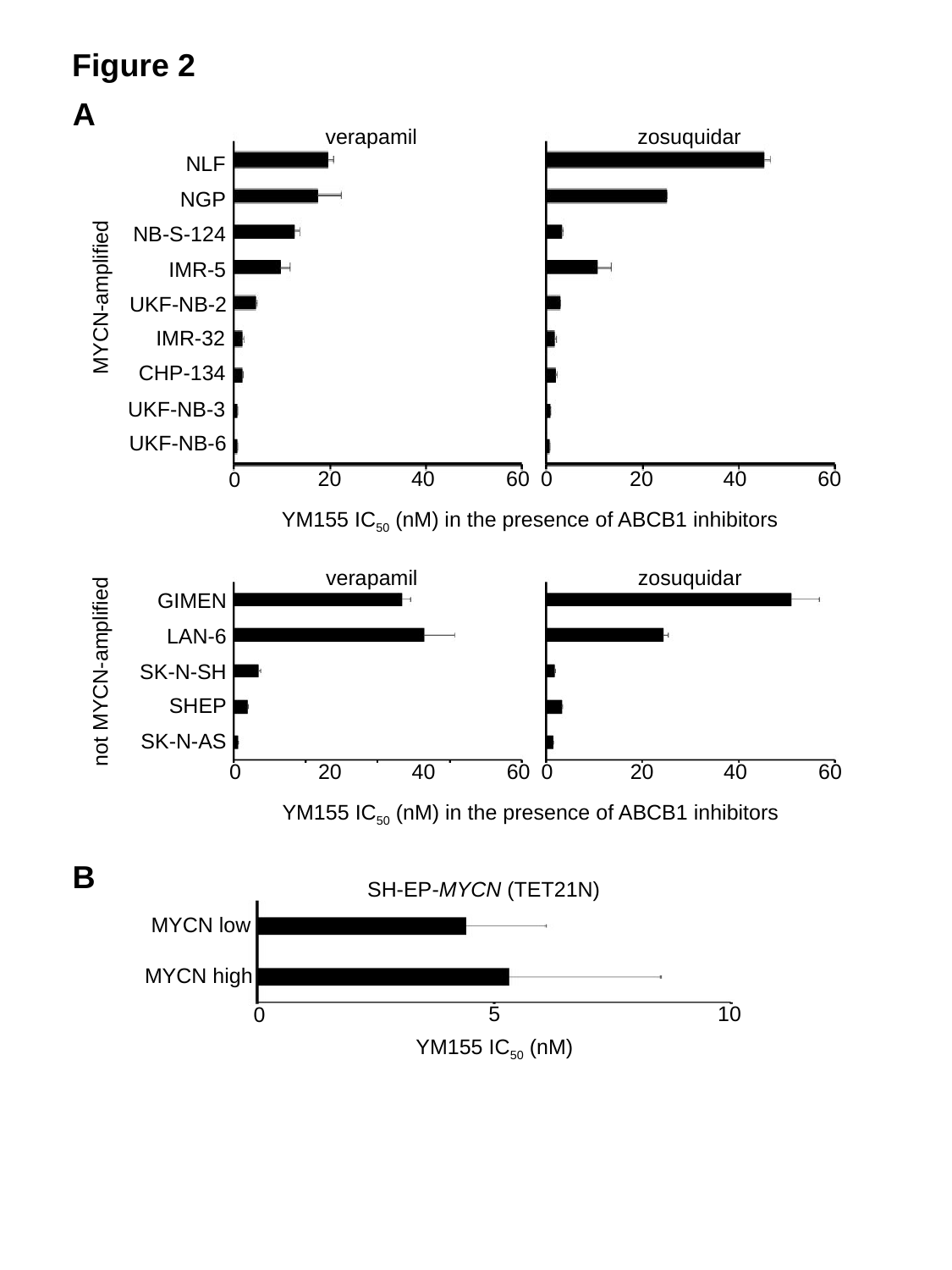

Figure 2
A
zosuquidar
verapamil
NLF
NGP
NB-S-124
IMR-5
MYCN-amplified
UKF-NB-2
IMR-32
CHP-134
UKF-NB-3
UKF-NB-6
20
40
60
0
20
40
60
0
YM155 IC50 (nM) in the presence of ABCB1 inhibitors
zosuquidar
verapamil
GIMEN
LAN-6
SK-N-SH
not MYCN-amplified
SHEP
SK-N-AS
20
40
60
0
20
40
60
0
YM155 IC50 (nM) in the presence of ABCB1 inhibitors
B
SH-EP-MYCN (TET21N)
MYCN low
MYCN high
5
10
0
YM155 IC50 (nM)
