# Supplementary material for: Testing of the Survivin Suppressant YM155 in a Large Panel of Drug-Resistant Neuroblastoma Cell Lines
Source: Cancers (Basel). 2020 Mar 2;12(3):577. doi: 10.3390/cancers12030577 (PMC7139505; doi:10.3390/cancers12030577)
Supplement: Supplementary file 1 [file cancers-12-00577-s001.zip › Michaelis et al_Supplements/Michaelis et al_Figure 3_revised.pptx]

## Slide 1
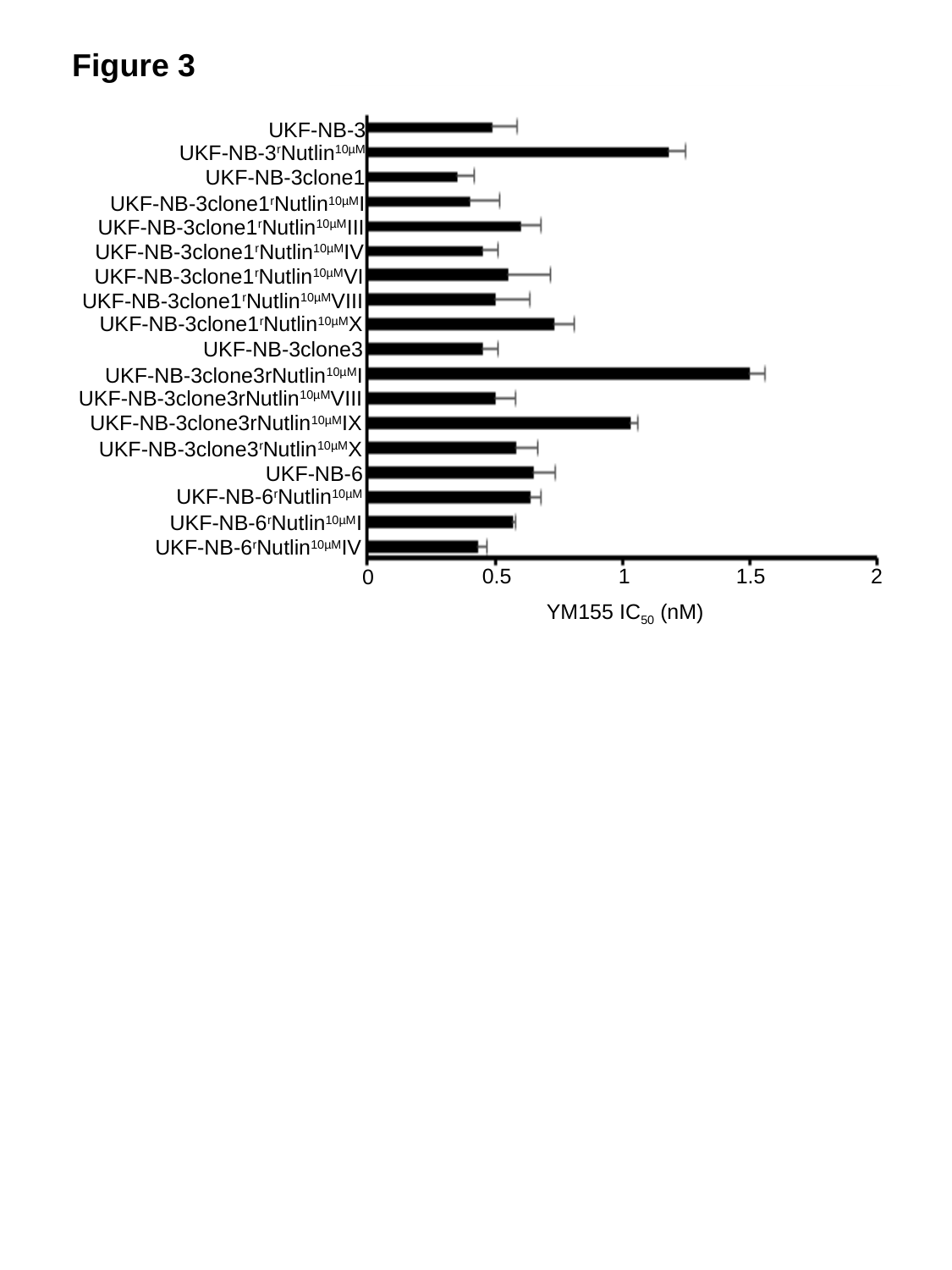

Figure 3
UKF-NB-3
UKF-NB-3rNutlin10µM
UKF-NB-3clone1
UKF-NB-3clone1rNutlin10µMI
UKF-NB-3clone1rNutlin10µMIII
UKF-NB-3clone1rNutlin10µMIV
UKF-NB-3clone1rNutlin10µMVI
UKF-NB-3clone1rNutlin10µMVIII
UKF-NB-3clone1rNutlin10µMX
UKF-NB-3clone3
UKF-NB-3clone3rNutlin10µMI
UKF-NB-3clone3rNutlin10µMVIII
UKF-NB-3clone3rNutlin10µMIX
UKF-NB-3clone3rNutlin10µMX
UKF-NB-6
UKF-NB-6rNutlin10µM
UKF-NB-6rNutlin10µMI
UKF-NB-6rNutlin10µMIV
0.5
1
1.5
2
0
YM155 IC50 (nM)
