# Supplementary material for: Testing of the Survivin Suppressant YM155 in a Large Panel of Drug-Resistant Neuroblastoma Cell Lines
Source: Cancers (Basel). 2020 Mar 2;12(3):577. doi: 10.3390/cancers12030577 (PMC7139505; doi:10.3390/cancers12030577)
Supplement: Supplementary file 1 [file cancers-12-00577-s001.zip › Michaelis et al_Supplements/Michaelis et al_Figure 4_revised.pptx]

## Slide 1
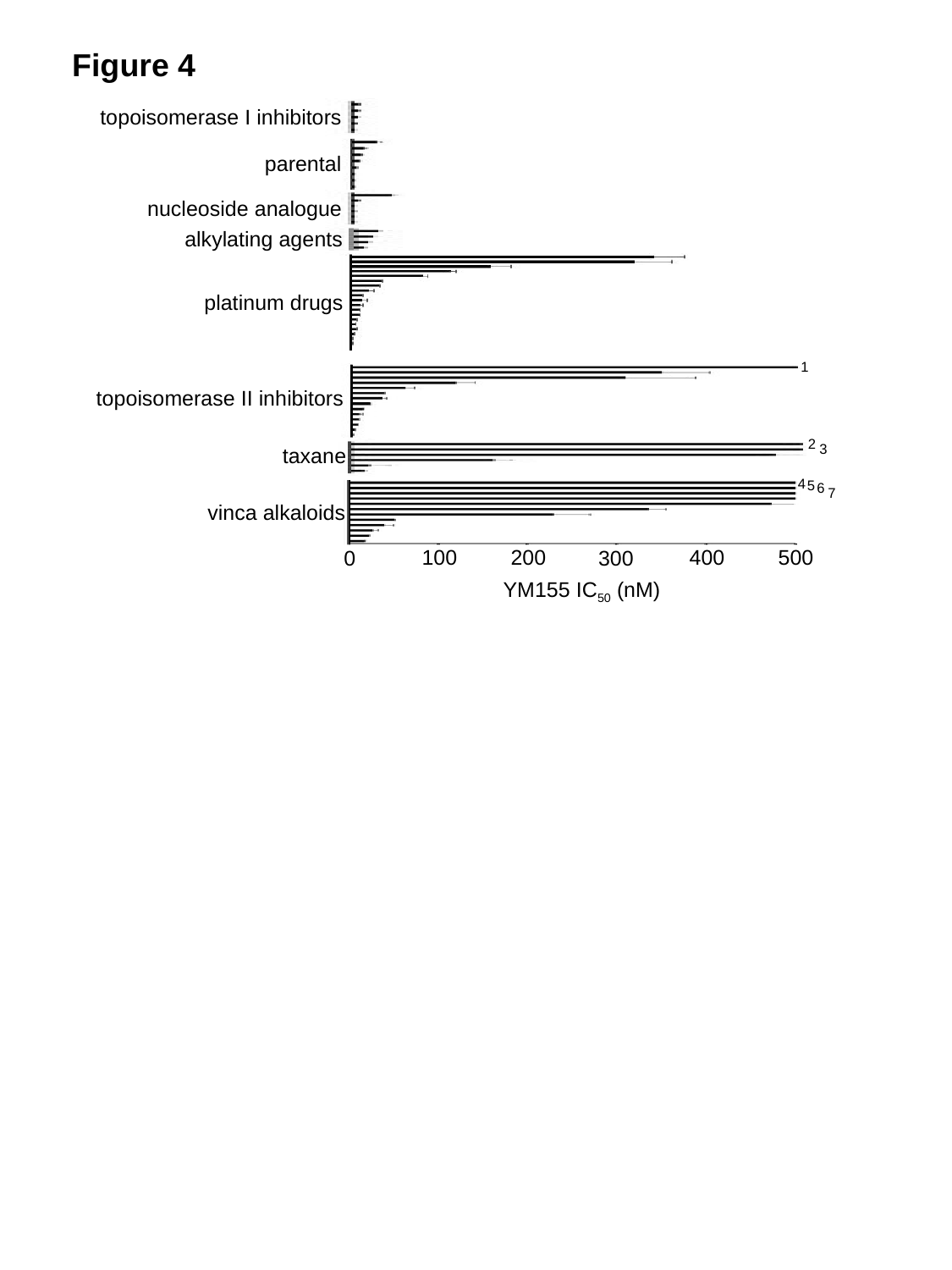

Figure 4
topoisomerase I inhibitors
parental
nucleoside analogue
alkylating agents
platinum drugs
1
topoisomerase II inhibitors
2
3
taxane
4
5
6
7
vinca alkaloids
100
400
200
500
0
300
YM155 IC50 (nM)
