# Supplementary material for: Testing of the Survivin Suppressant YM155 in a Large Panel of Drug-Resistant Neuroblastoma Cell Lines
Source: Cancers (Basel). 2020 Mar 2;12(3):577. doi: 10.3390/cancers12030577 (PMC7139505; doi:10.3390/cancers12030577)
Supplement: Supplementary file 1 [file cancers-12-00577-s001.zip › Michaelis et al_Supplements/Michaelis et al_Figure S1_revised.pdf]

Figure S1

A

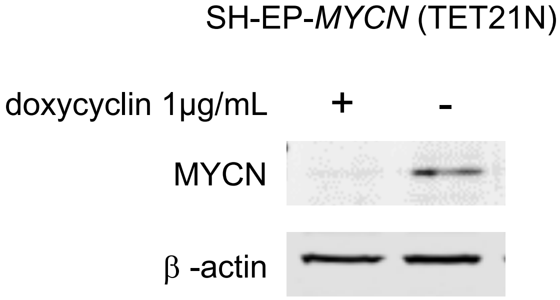

B

	YM155 IC_{50} (nM)
- doxycyclin (1µg/mL)	5.31 ± 3.21
+ doxycyclin (1µg/mL)	4.40 ± 1.69

Figure S1. Effects of MYCN expression on neuroblastoma cell sensitivity to YM155. A) MYCN levels in SH-EP-*MYCN* (TET21N) cells in the absence or presence of doxycycline; B) YM155 concentrations that reduce the viability of SH-EP-*MYCN* (TET21N) cells by 50% (IC_{50}, mean ± S.D., n = 3) in the absence or presence of doxycycline as determined by MTT assay after a 5-day treatment period.

Figure S1

Uncropped blots

MYCN

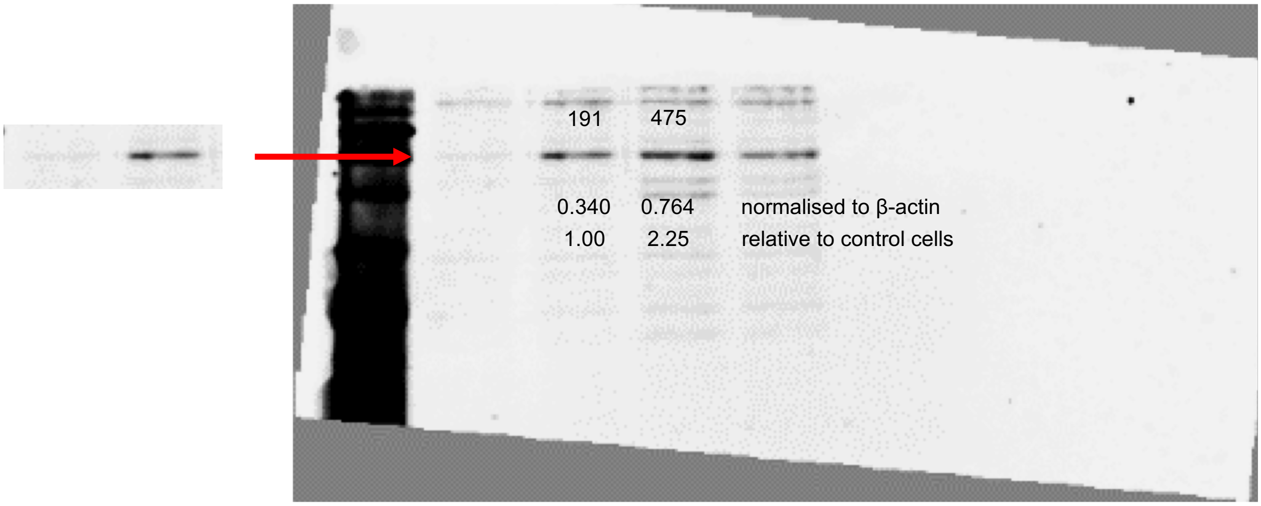

β-actin

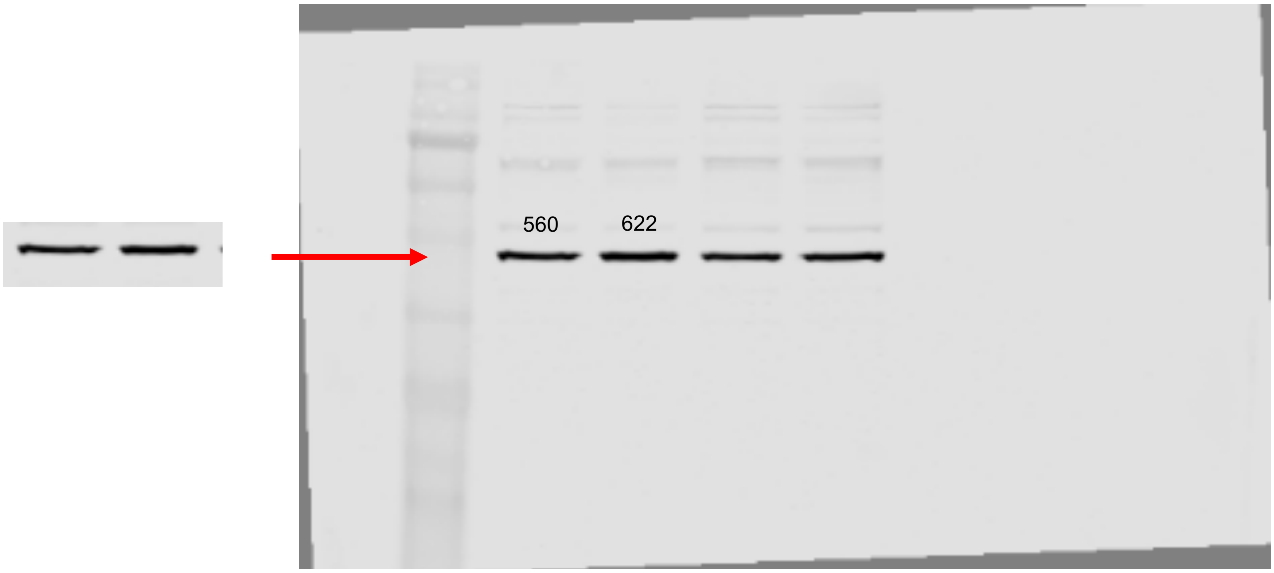

Densitometric analysis was performed with QuantiOne (BioRad). MYCN levels were normalised to β-actin expression and values relative to control cells are displayed.
