# Supplementary material for: Testing of the Survivin Suppressant YM155 in a Large Panel of Drug-Resistant Neuroblastoma Cell Lines
Source: Cancers (Basel). 2020 Mar 2;12(3):577. doi: 10.3390/cancers12030577 (PMC7139505; doi:10.3390/cancers12030577)
Supplement: Supplementary file 1 [file cancers-12-00577-s001.zip › Michaelis et al_Supplements/Michaelis et al_Figure S2_revised.pdf]

Figure S2

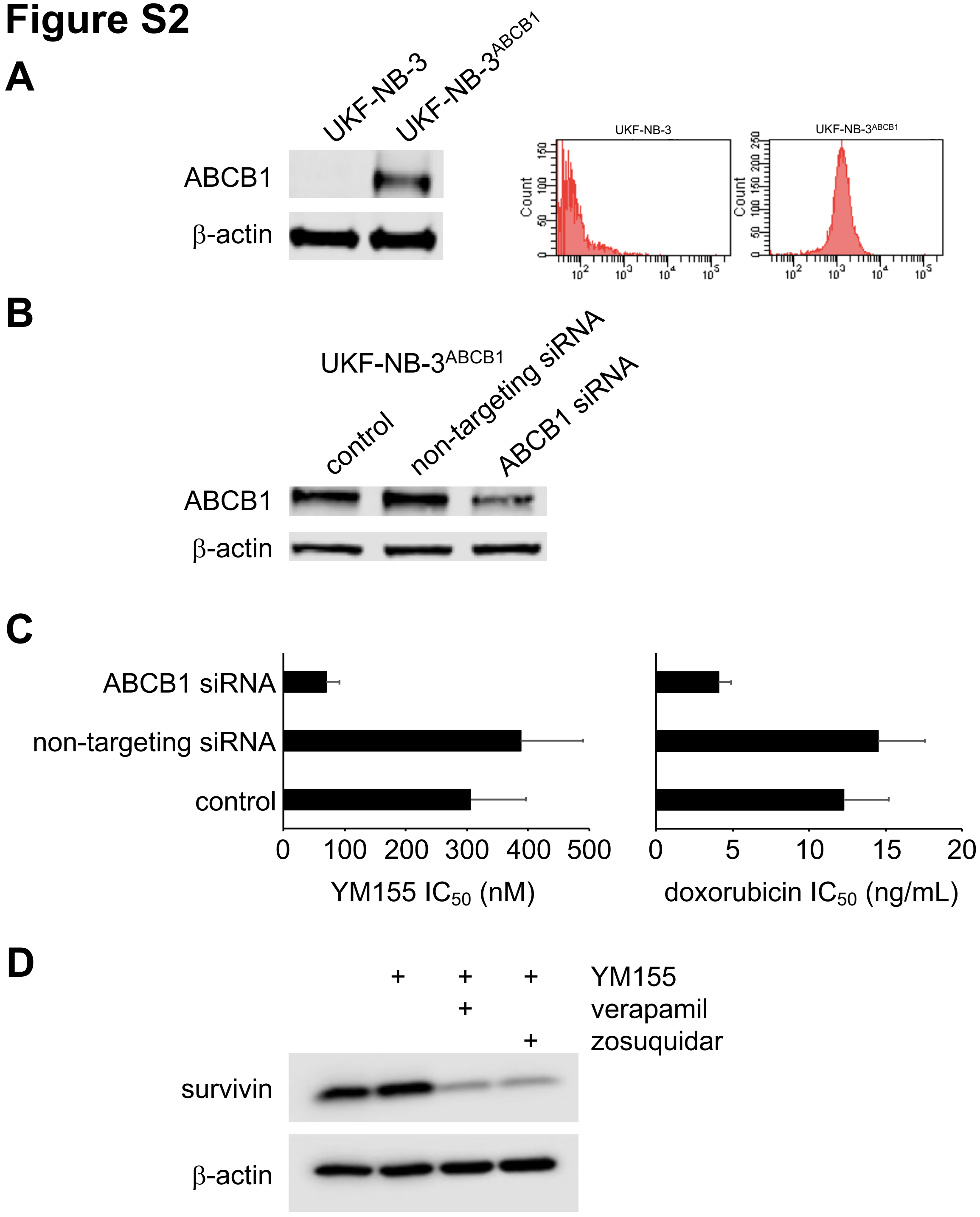

Figure S2. Effects of YM155 in ABCB1-transduced cells. A) Representative Western blots and flow cytometry histograms indicating ABCB1 levels in UKF-NB-3 cells and in UKF-NB-3 transduced with a lentiviral vector encoding ABCB1 (UKF-NB-3^{ABCB1}) . B) Effect of siRNA directed against ABCB1 on cellular ABCB1 levels in UKF-NB-3^{ABCB1} cells. C) Concentrations of YM155 and doxorubicin (alternative ABCB1 substrate used as control) that reduce the viability of UKF-NB-3^{ABCB1} cells by 50% (IC_{50}, mean ± S.D., n = 3) as determined by MTT assay after 120h of incubation. D) Effects of YM155 (100nM) on survivin levels in UKF-NB-3^{ABCB1} cells after 24h of incubation in the presence or absence of verapamil (5μM) or zosuquidar (1.25μM).

Figure S2

Uncropped blots S2A

ABCB1

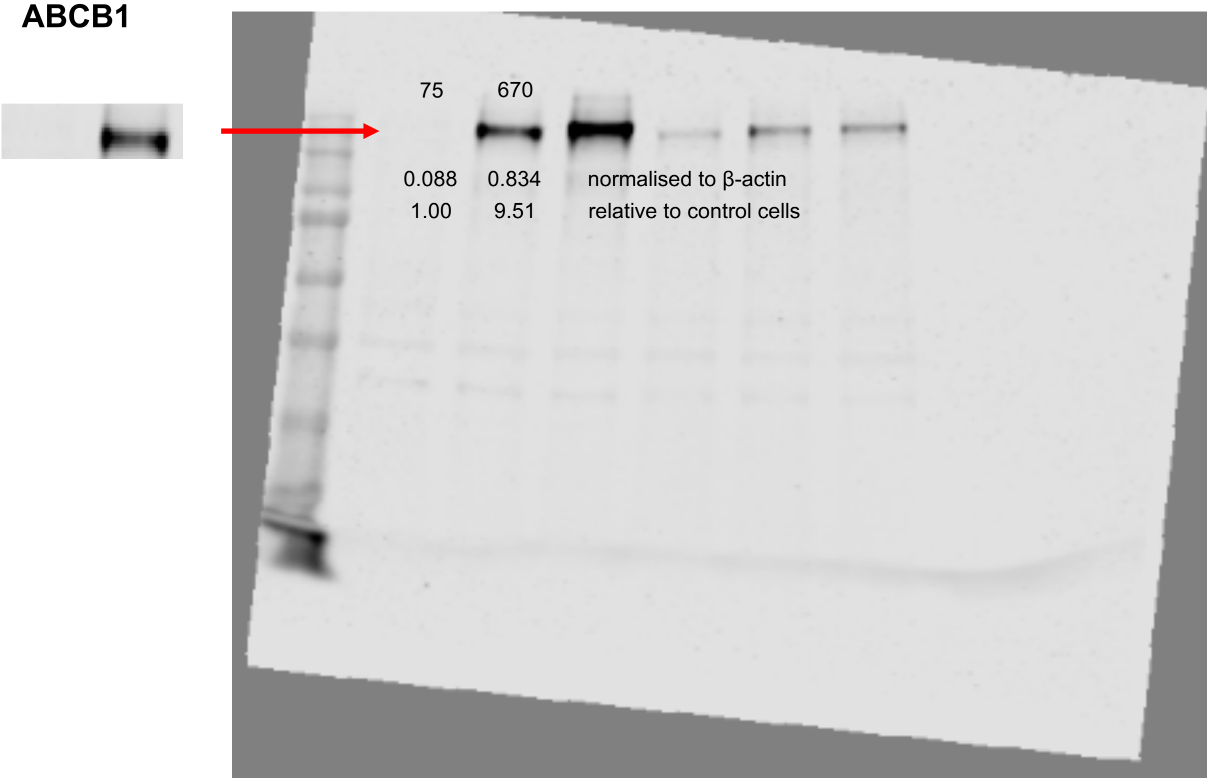

β-actin

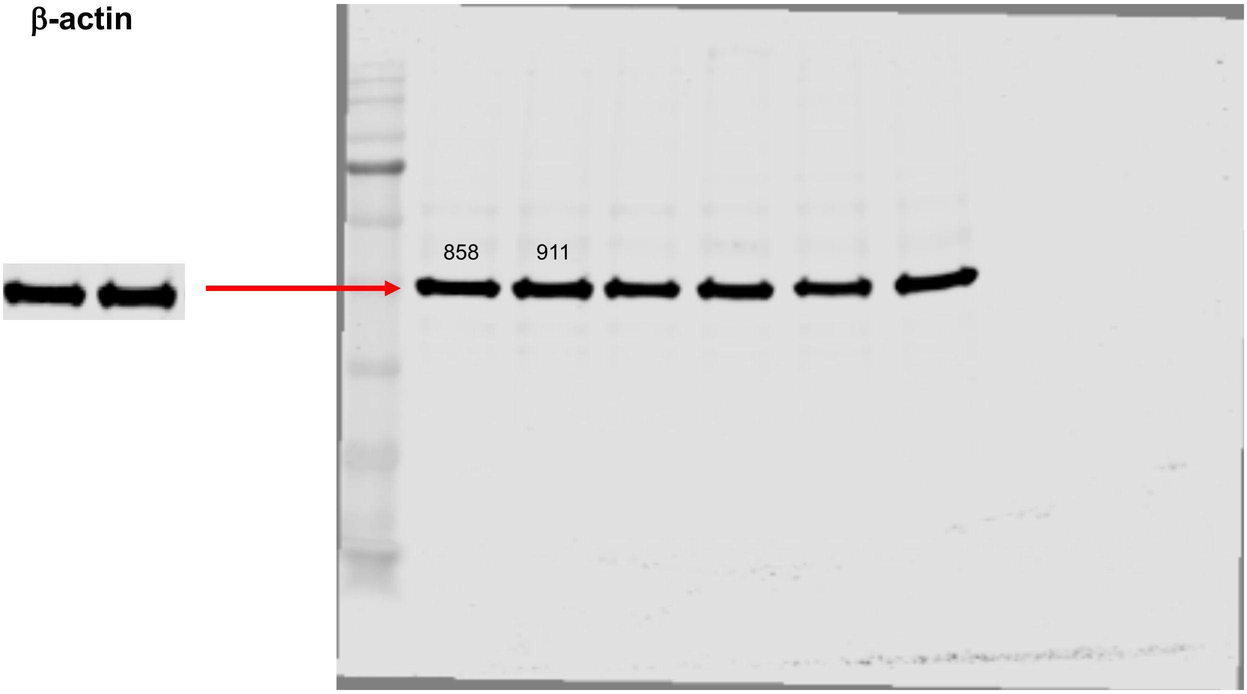

Densitometric analysis was performed with QuantiOne (BioRad). ABCB1 levels were normalised to β-actin expression and values relative to control cells are displayed.

Figure S2

Uncropped blots S2B

ABCB1

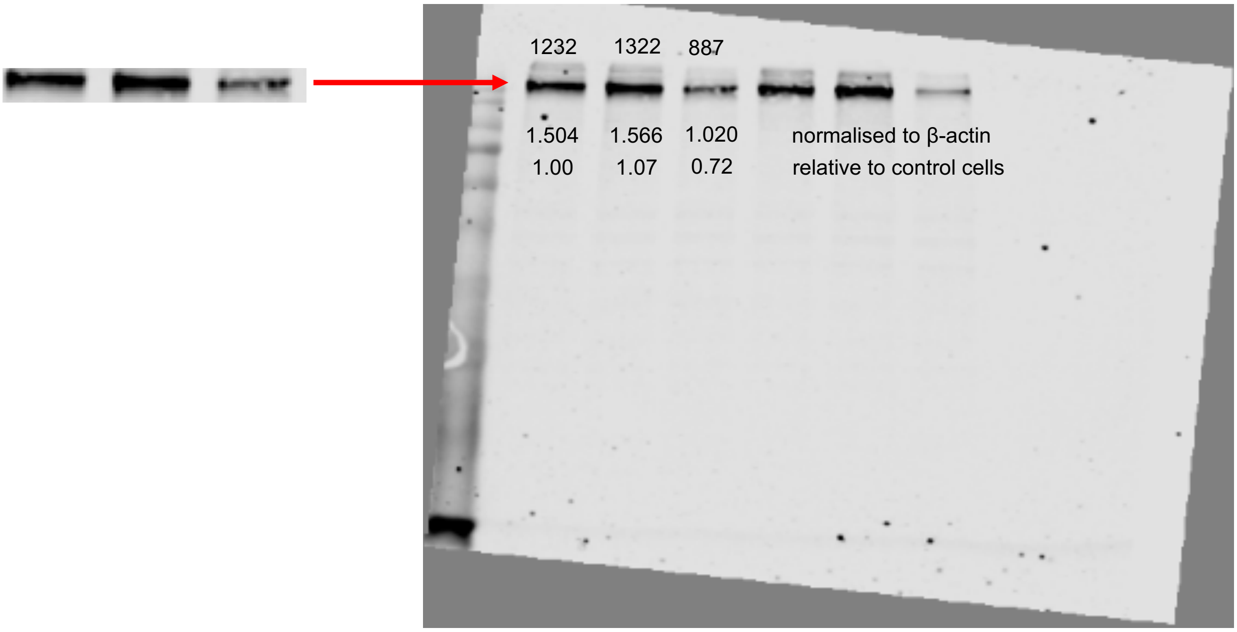

β-actin

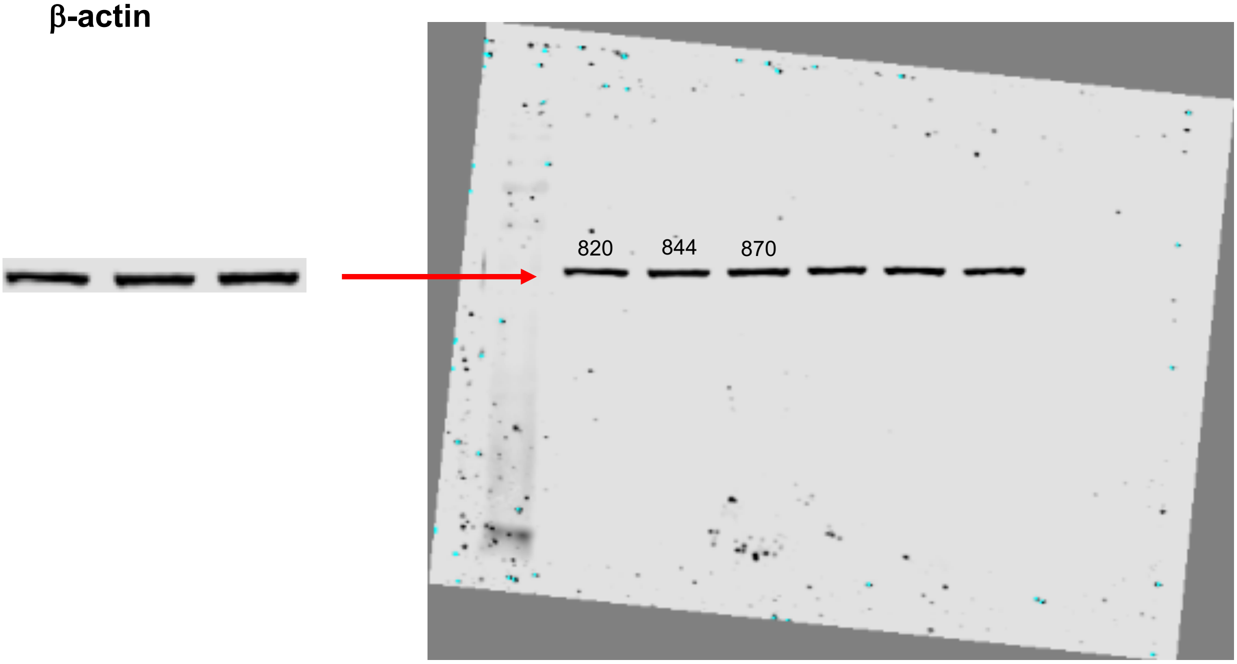

Densitometric analysis was performed with QuantiOne (BioRad). ABCB1 levels were normalised to β-actin expression and values relative to control cells are displayed.

Figure S2

Uncropped blots S2D

survivin

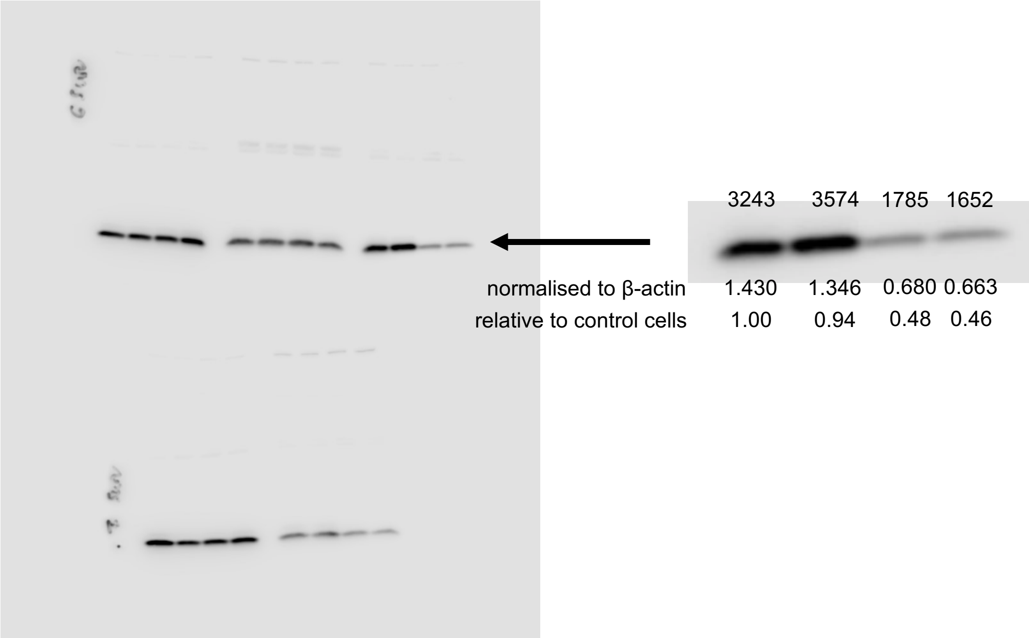

β-actin

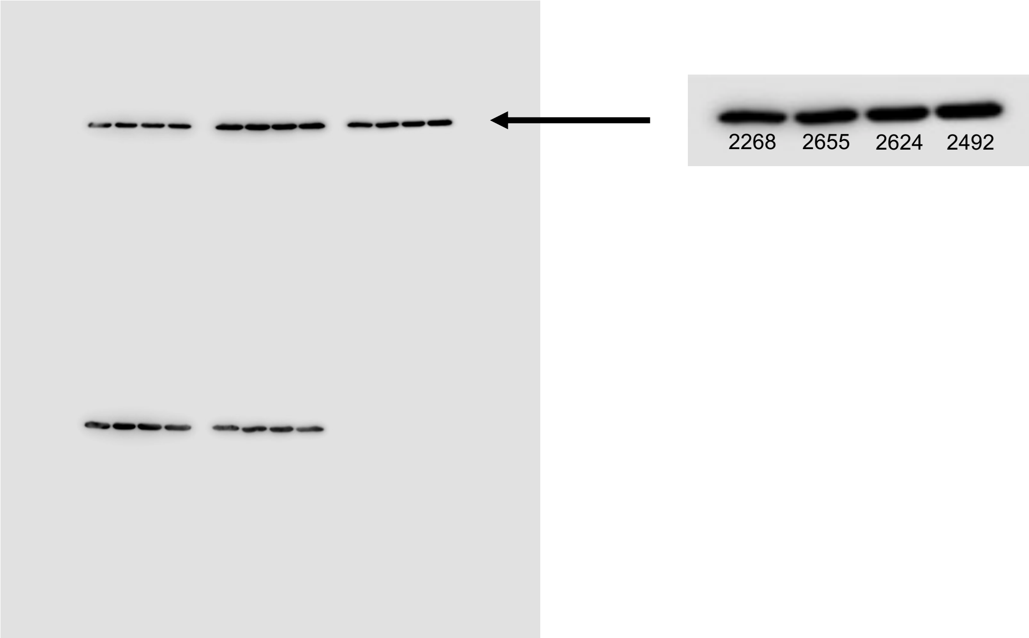

Densitometric analysis was performed with QuantiOne (BioRad). Survivin levels were normalised to β-actin expression and values relative to control cells are displayed.
