# Supplementary material for: Testing of the Survivin Suppressant YM155 in a Large Panel of Drug-Resistant Neuroblastoma Cell Lines
Source: Cancers (Basel). 2020 Mar 2;12(3):577. doi: 10.3390/cancers12030577 (PMC7139505; doi:10.3390/cancers12030577)
Supplement: Supplementary file 1 [file cancers-12-00577-s001.zip › Michaelis et al_Supplements/Michaelis et al_Table S7_revised_02.pdf]

Table S7. YM155 concentrations that reduce the viability of neuroblastoma cell lines by 50% (IC_{50}, mean ± S.D., n = 3) in the absence or presence of zosuquidar (1.25μM) as indicated by MTT assay or CellTiterGlo after 120h of incubation.

**Cell line**	**YM155 IC_{50} (nM)**	**+ Zosuquidar[1]**
IMR-5 (MTT)	7.18 ± 1.04	10.64 ± 2.80
IMR-5 (CellTiterGlo)	6.27 ± 1.56	19.99 ± 5.97
IMR-5^{r}DOCE^{20} (MTT)	21,549 ± 638	13.63 ± 5.54
IMR-5^{r}DOCE^{20} (CellTiterGlo)	32,946 ± 4360	14.82 ± 4.66
IMR-32 (MTT)	1.40 ± 0.35	1.80 ± 0.23
IMR-32 (CellTiterGlo)	1.22 ± 0.21	1.91 ± 1.16
IMR-32^{r}DOX^{20} (MTT)	35.53 ± 2.23	0.94 ± 0.08
IMR-32^{r}DOX^{20} (CellTiterGlo)	29.20 ± 3.56	0.86 ± 0.06

[1] The effects of zosuquidar alone are provided in Table S5.
