# Supplementary material for: Testing of the Survivin Suppressant YM155 in a Large Panel of Drug-Resistant Neuroblastoma Cell Lines
Source: Cancers (Basel). 2020 Mar 2;12(3):577. doi: 10.3390/cancers12030577 (PMC7139505; doi:10.3390/cancers12030577)
Supplement: Supplementary file 1 [file cancers-12-00577-s001.zip › Michaelis et al_Supplements/Michaelis et al_Table S8_revised_02.pdf]

Table S8. YM155 concentrations that reduce the viability of neuroblastoma cell lines by 50% (IC_{50}, mean ± S.D., n = 3) in the absence or presence of the ABCC1 inhibitor MK571 (10μM) as indicated by MTT assay after 120h of incubation.

		**+ MK571**	
**Cell line**	**YM155 IC_{50} (nM)**	**MK571 alone[1]**	**YM155 IC_{50} (nM)**
NLF	26.3 ± 5.9	98 ± 15	25.6 ± 9.1
NLF^{r}VCR[10]	324 ± 79	102 ± 17	141 ± 38

[1] Effect of MK571 (10μM) on cell viability in percentage relative to untreated control.
