# Supplementary material for: Testing of the Survivin Suppressant YM155 in a Large Panel of Drug-Resistant Neuroblastoma Cell Lines
Source: Cancers (Basel). 2020 Mar 2;12(3):577. doi: 10.3390/cancers12030577 (PMC7139505; doi:10.3390/cancers12030577)
Supplement: Supplementary file 1 [file cancers-12-00577-s001.zip › Michaelis et al_Supplements/Michaelis et al_Table S1_revised_02.pdf]

Table S1. YM155 concentrations that reduce the viability of neuroblastoma cell lines by 50% (IC_{50}, mean ± S.D., n = 3) as indicated by MTT assay after 120h of incubation.

**Cell line**	**YM155 IC_{50} (nM)**	**Cell line**	**YM155 IC_{50} (nM)**
Be(2)-C	24.25 ± 2.59	NLF^{r}GEMCI^{20}	1.84 ± 0.47 (0.1)
CHP-134	2.64 ± 0.50	NLF^{r}IRINO^{1000}	6.93 ± 0.71 (0.3)
GIMEN	33.74 ± 2.26	NLF^{r}MEL^{3000}	15.36 ± 3.20 (0.6)
IMR-5	7.18 ± 1.04	NLF^{r}OXALI^{4000}	33.67 ± 2.67 (1.3)
IMR-5^{r}CARBO^{5000} [1]	8.55 ± 2.01 (1.2)[2]	NLF^{r}VCR^{10}	334.5 ± 21.6 (12.5)
IMR-5^{r}CDDP^{1000}	19.71 ± 5.70 (2.7)	NLF^{r}VINB^{10}	38.10 ± 12.02 (1.4)
IMR-5^{r}DOCE^{20}	21549 ± 638 (3001)	NMB	6.41 ± 1.17
IMR-5^{r}DOX^{20}	116.3 ± 21.6 (16.2)	SHEP	10.15 ± 0.84
IMR-5^{r}ETO^{100}	8.29 ± 3.95 (1.2)	SHEP^{r}CDDP^{1000}	30.83 ± 2.24 (13.7)
IMR-5^{r}GEMCI^{20}	7.08 ± 1.20 (1.0)	SHEP^{r}ETO^{100}	20.24 ± 10.16 (2.0)
IMR-5^{r}MEL^{1000}	11.10 ± 1.57 (1.5)	SHEP^{r}VCR^{10}	20.95 ± 1.45 (2.1)
IMR-5^{r}OXALI^{4000}	10.18 ± 2.69 (1.4)	SH-SY5Y	31.8 ± 6.50
IMR-5^{r}TOPO^{20}	4.88 ± 1.72 (0.7)	SK-N-AS	3.55 ± 0.21
IMR-5^{r}VCR^{10}	472.9 ± 97.4 (65.9)	SK-N-SH	74.94 ± 19.52
IMR-5^{r}VINB^{20}	1608 ± 212 (224)	UKF-NB-2	4.18 ± 0.27
IMR-5^{r}VINOR^{20}	4978 ± 147 (693)	UKF-NB-2^{r}CARBO^{2000}	318.2 ± 42.7 (76.1)
IMR-32	1.40 ± 0.35	UKF-NB-2^{r}CDDP^{1000}	1.15 ± 1.21 (0.3)
IMR-32^{r}CARBO^{1000}	9.35 ± 0.97	UKF-NB-2^{r}DOCE^{10}	1108 ± 179 (265)
IMR-32^{r}DOX^{20}	35.63 ± 2.23 (5.0)	UKF-NB-2^{r}DOX^{20}	347.0 ± 55.2 (83.0)
IMR-32^{r}ETO^{100}	1.53 ± 0.13 (0.2)	UKF-NB-2^{r}OXALI^{600}	3.25 ± 0.64 (0.8)
IMR-32^{r}GEMCI^{20}	2.16 ± 0.22 (0.3)	UKF-NB-2^{r}VCR^{10}	5940 ± 247 (1421)
IMR-32^{r}OXALI^{800}	0.60 ± 0.02 (0.1)	UKF-NB-3	0.49 ± 0.10
IMR-32^{r}TOPO$^{7.5}$	0.45 ± 0.06 (0.1)	UKF-NB-3^{r}CARBO^{2000}	155.4 ± 24.6 (317)
IMR-32^{r}VINOR^{5}	16.43 ± 1.08 (2.3)	UKF-NB-3^{r}CDDP^{1000}	5.32 ± 1.21 (10.9)
LAN-6	248.1 ± 32.9	UKF-NB-3^{r}DOCE^{10}	469.6 ± 113.1 (958)
NB-S-124	76.66 ± 6.51	UKF-NB-3^{r}DOX^{20}	15,700 ± 1,019 (32041)
NGP	12.48 ± 3.01	UKF-NB-3^{r}ETO^{200}	7.97 ± 0.13 (16.3)
NGP^{r}CARBO^{5000}	112.3 ± 5.0 (9.0)	UKF-NB-3^{r}GEMCI^{10}	0.40 ± 0.01 (0.8)
NGP^{r}CDDP^{1000}	13.00 ± 0.42 (1.0)	UKF-NB-3^{r}Nutlin$^{10 \mu M}$	1.18 ± 0.07 (2.4)
NGP^{r}DACARB^{18}	20.59 ± 1.84 (1.6)	UKF-NB-3^{r}OXALI^{4000}	1.80 ± 0.78 (3.7)
NGP^{r}DOCE^{20}	159.0 ± 19.5 (12.7)	UKF-NB-3^{r}TOPO^{20}	7.40 ± 0.71 (15.1)
NGP^{r}DOX^{20}	306.9 ± 78.5 (24.6)	UKF-NB-3^{r}VCR^{10}	26.59 ± 6.37 (54.3)
NGP^{r}ETO^{400}	59.20 ± 11.40 (4.7)	UKF-NB-6	0.65 ± 0.09
NGP^{r}GEMCI^{20}	41.55 ± 6.13 (3.3)	UKF-NB-6^{r}CARBO^{2000}	16.83 ± 1.62 (25.9)
NGP^{r}MEL^{3000}	26.10 ± 3.86 (2.1)	UKF-NB-6^{r}CDDP^{2000}	79.93 ± 7.14 (123)
NGP^{r}OXALI^{4000}	6.93 ± 0.28 (0.6)	UKF-NB-6^{r}DOCE^{10}	14.33 ± 4.08 (22.0)
NGP^{r}VCR^{20}	6986 ± 715 (560)	UKF-NB-6^{r}DOX^{20}	11.80 ± 1.56 (18.2)
NLF	26.78 ± 4.04	UKF-NB-6^{r}ETO^{200}	3.60 ± 0.01 (5.5)
NLF^{r}CARBO^{5000}	340.5 ± 34.5 (12.7)	UKF-NB-6^{r}GEMCI^{10}	2.10 ± 0.84 (3.2)
NLF^{r}CDDP^{500}	12.58 ± 5.39 (0.5)	UKF-NB-6^{r}OXALI^{4000}	5.34 ± 0.71 (8.2)
NLF^{r}DOCE^{20}	21.6 ± 5.98 (0.8)	UKF-NB-6^{r}TOPO^{20}	3.47 ± 0.81 (5.3)
NLF^{r}DOX^{40}	34.88 ± 4.33 (1.3)	UKF-NB-6^{r}VCR^{10}	49.30 ± 2.24 (75.8)
NLF^{r}ETO^{100}	7.40 ± 0.54 (0.3)	UKF-NB-6^{r}VINOR^{40}	228.5 ± 41.5 (352)

[1] CARBO, carboplatin; CDDP, cisplatin; DACARB, dacarbazine; DOX, doxorubicin; ETO, etoposide; GEMCI, gemcitabine; IRINO, irinotecan; MEL, melphalan; Nutlin, nutlin-3; OXALI, oxaliplatin; TOPO, topotecan; VCR, vincristine; VINB, vinblastine; VINOR, vinorelbine

[2] fold difference IC_{50} resistant sub-line/ IC_{50} respective parental cell line
