# Supplementary material for: Testing of the Survivin Suppressant YM155 in a Large Panel of Drug-Resistant Neuroblastoma Cell Lines
Source: Cancers (Basel). 2020 Mar 2;12(3):577. doi: 10.3390/cancers12030577 (PMC7139505; doi:10.3390/cancers12030577)
Supplement: Supplementary file 1 [file cancers-12-00577-s001.zip › Michaelis et al_Supplements/Michaelis et al_Table S6_revised_02.pdf]

Table S6. YM155 concentrations that reduce the viability of neuroblastoma cell lines by 50% (IC$_{50}$, mean ± S.D., n = 3) in the absence or presence of the ABCB1 inhibitors verapamil (5 µM) or zosuquidar (1.25 µM) as indicated by MTT assay after 120h of incubation.

		**+ verapamil (5 µM)**		**+ zosuquidar (1.25 µM)**	
**Cell line**	**YM155 IC$_{50}$ (nM)**	**verapamil alone**	**YM155 IC$_{50}$ (nM)**	**zosuquidar alone**	**YM155 IC$_{50}$ (nM)**
CHP-134	*2.64 ± 0.50*	*94 ± 13*[1]	*1.64 ± 0.27 (1.6)*[2]	*105 ± 6*[1]	*1.85 ± 0.34 (2.4)*
GIMEN	*33.74 ± 2.26*	*105 ± 3*	*52.90 ± 8.62 (0.6)*	*92 ± 7*	*50.87 ± 5.91 (0.7)*
IMR-5	*7.18 ± 1.04*	*109 ± 8*	*9.70 ± 1.97 (0.7)*	*104 ± 11*	*10.64 ± 2.80 (0.7)*
IMR-5^{r}CARBO^{5000}	8.55 ± 2.01	91 ± 16	7.80 ± 0.28 (1.1)	105 ± 8	27.01 ± 3.04 (0.3)
IMR-5^{r}CDDP^{1000}	19.71 ± 5.70	88 ± 11	15.23 ± 4.21 (1.3)	100 ± 6	33.47 ± 6.84 (0.6)
IMR-5^{r}DOCE^{20}	21549 ± 638	90 ± 7	149.01 ± 1.99 (145)	112 ± 2	13.63 ± 5.54 (1581)
IMR-5^{r}DOX^{20}	116.31 ± 21.63	97 ± 9	17.60 ± 0.57 (6.6)	99 ± 8	13.45 ± 2.45 (8.6)
IMR-5^{r}ETO^{100}	8.29 ± 3.95	95 ± 10	6.99 ± 2.79 (1.2)	97 ± 4	18.26 ± 3.19 (0.5)
IMR-5^{r}GEMCI^{20}	7.08 ± 1.20	108 ± 6	7.90 ± 2.09 (0.9)	105 ± 8	12.73 ± 3.34 (0.6)
IMR-5^{r}MEL^{1000}	11.10 ± 1.57	92 ± 8	6.63 ± 1.30 (1.7)	107 ± 6	12.80 ± 1.22 (0.9)
IMR-5^{r}OXALI^{4000}	10.18 ± 2.69	96 ± 8	15.80 ± 1.77 (0.6)	110 ± 6	16.81 ± 2.71 (0.6)
IMR-5^{r}TOPO^{20}	4.88 ± 1.72	100 ± 13	5.94 ± 1.31 (0.8)	101 ± 7	11.77 ± 3.95 (0.4)
IMR-5^{r}VCR^{10}	472.94 ± 97.42	93 ± 8	13.05 ± 2.90 (36)	94 ± 5	19.35 ± 0.07 (24)
IMR-5^{r}VINB^{20}	1608 ± 212	93 ± 7	9.34 ± 0.94 (172)	93 ± 6	10.05 ± 1.06 (160)
IMR-32	*1.40 ± 0.35*	*102 ± 7*	*1.70 ± 0.41 (0.8)*	*101 ± 3*	*1.80 ± 0.23 (0.8)*
IMR-32^{r}DOX^{20}	35.63 ± 2.23	92 ± 15	1.75 ± 0.77 (20)	89 ± 13	0.94 ± 0.08 (38)
IMR-32^{r}ETO^{100}	1.53 ± 0.13	90 ± 5	1.60 ± 0.27 (1.0)	104 ± 6	3.55 ± 0.21 (2.2)
IMR-32^{r}GEMCI^{20}	2.16 ± 0.22	105 ± 8	1.15 ± 0.05 (1.9)	107 ± 11	4.20 ± 0.45 (0.5)
IMR-32^{r}OXALI^{800}	0.60 ± 0.02	99 ± 8	0.71 ± 0.08 (0.8)	105 ± 12	1.18 ± 0.07 (1.7)
IMR-32^{r}TOPO$^{7.5}$	0.45 ± 0.06	97 ± 10	0.61 ± 0.07 (0.7)	101 ± 2	0.97 ± 0.04 (0.5)
LAN-6	*248.08 ± 32.90*	*99 ± 8*	*46.75 ± 2.33 (5.3)*	*103 ± 5*	*24.35 ± 1.06 (10.2)*
NB-S-124	*76.66 ± 6.51*	*103 ± 6*	*12.52 ± 1.16 (6.1)*	*110 ± 8*	*3.20 ± 0.40 (24.0)*
NGP	*12.48 ± 3.01*	*91 ± 8*	*17.35 ± 4.97 (0.7)*	*109 ± 2*	*24.95 ± 0.21 (0.5)*
NGP^{r}CARBO^{5000}	112.33 ± 5.0	112 ± 9	76.10 ± 3.17 (1.5)	107 ± 5	158.24 ± 9.34 (0.7)
NGP^{r}CDDP^{1000}	13.00 ± 0.42	104 ± 5	19.61 ± 1.35 (0.7)	101 ± 18	17.80 ± 0.97 (0.7)
NGP^{r}DACARB^{18}	20.59 ± 1.84	107 ± 2	26.26 ± 4.77 (0.8)	103 ± 7	41.90 ± 5.27 (0.5)
NGP^{r}DOX^{20}	306.90 ± 78.5	92 ± 6	5.52 ± 0.35 (56)	90 ± 2	0.70 ± 0.04 (438)
NGP^{r}ETO^{400}	59.20 ± 11.40	98 ± 16	50.14 ± 16.45 (1.2)	98 ± 3	39.12 ± 7.87 (1.5)
NGP^{r}GEMCI^{20}	41.55 ± 6.13	94 ± 13	73.43 ± 16.41 (0.6)	105 ± 12	10.50 ± 1.34 (4.0)
NGP^{r}MEL^{3000}	26.10 ± 3.86	99 ± 10	24.34 ± 1.76 (1.1)	108 ± 9	18.75 ± 4.64 (1.4)
NGP^{r}OXALI^{4000}	6.93 ± 0.28	102 ± 8	12.25 ± 2.78 (0.6)	101 ± 12	8.21 ± 1.04 (0.8)
NGP^{r}VCR^{20}	6986.25 ± 715	100 ± 10	157.60 ± 11.79 (44)	106 ± 15	16.20 ± 1.74 (431)
NLF	*4.18 ± 0.27*	*93 ± 8*	*4.55 ± 0.32 (0.9)*	*99 ± 5*	*2.85 ± 0.14 (1.5)*

NLF^{r}CARBO^{5000}	340.51 ± 34.47	95 ± 7	196.4 ± 17.9 (1.7)	104 ± 8	569.90 ± 76.7 (0.6)
NLF^{r}CDDP^{500}	12.58 ± 5.39	103 ± 6	20.10 ± 1.84 (0.6)	101 ± 15	24.84 ± 3.25 (0.5)
NLF^{r}DOCE^{200}	21.60 ± 5.98	99 ± 4	8.58 ± 0.76 (2.5)	109 ± 9	3.93 ± 0.17 (5.5)
NLF^{r}DOX^{40}	34.88 ± 4.33	110 ± 18	31.15 ± 0.78 (1.1)	98 ± 6	59.81 ± 1.41 (0.6)
NLF^{r}ETO^{100}	7.40 ± 0.54	108 ± 5	9.35 ± 0.50 (0.8)	97 ± 12	11.05 ± 0.63 (0.7)
NLF^{r}GEMCI^{20}	1.84 ± 0.47	94 ± 10	2.30 ± 0.14 (0.8)	108 ± 13	2.65 ± 0.50 (0.7)
NLFr^{I}RINO^{1000}	6.93 ± 0.71	98 ± 9	3.83 ± 0.64 (1.8)	89 ± 17	10.96 ± 1.37 (0.6)
NLF^{r}MEL^{3000}	15.36 ± 3.20	100 ± 7	12.70 ± 0.14 (1.2)	105 ± 11	27.45 ± 0.99 (0.6)
NLF^{r}OXALI^{4000}	33.67 ± 2.67	102 ± 11	30.05 ± 2.33 (1.1)	96 ± 3	51.25 ± 13.51 (0.7)
NLF^{r}VCR^{10}	334.45 ± 21.6	90 ± 14	74.70 ± 19.13 (4.5)	99 ± 18	295.8 ± 34.9 (1.1)
NLF^{r}VINB^{10}	38.10 ± 12.02	95 ± 6	36.74 ± 7.24 (1.0)	106 ± 8	65.67 ± 11.06 (0.6)
SHEP	*10.15 ± 0.84*	*88 ± 5*	*3.92 ± 0.11 (2.6)*	*95 ± 11*	*3.20 ± 0.14 (3.2)*
SHEP^{r}CDDP^{1000}	30.83 ± 2.24	105 ± 3	5.50 ± 0.37 (5.6)	110 ± 8	5.27 ± 0.17 (5.9)
SHEP^{r}ETO^{100}	20.24 ± 10.16	101 ± 14	5.35 ± 0.17 (3.8)	103 ± 12	5.11 ± 0.83 (4.0)
SHEP^{r}VCR^{10}	20.95 ± 1.45	99 ± 9	8.31 ± 1.55 (2.5)	107 ± 13	3.64 ± 0.09 (5.8)
SK-N-AS	*3.55 ± 0.21*	*107 ± 3*	*1.01 ± 0.26 (3.5)*	*97 ± 9*	*1.31 ± 0.11 (2.7)*
SK-N-SH	*74.94 ± 19.52*	*91 ± 12*	*6.80 ± 0.83 (11.0)*	*94 ± 6*	*1.72 ± 0.15 (43.6)*
UKF-NB-2	*4.18 ± 0.27*	*93 ± 8*	*4.55 ± 0.32 (0.9)*	*99 ± 5*	*2.85 ± 0.14 (1.5)*
UKF-NB-2^{r}CARBO^{2000}	318.21 ± 42.68	106 ± 14	114.7 ± 14.6 (2.8)	113 ± 19	7.67 ± 1.29 (41.5)
UKF-NB-2^{r}OXALI^{600}	3.25 ± 0.64	89 ± 10	5.90 ± 0.97 (0.6)	109 ± 16	3.00 ± 0.73 (1.1)
UKF-NB-3	*0.49 ± 0.10*	*90 ± 9*	*0.61 ± 0.13 (0.8)*	*102 ± 6*	*0.74 ± 0.10 (0.7)*
UKF-NB-3^{r}CARBO^{2000}	155.36 ± 24.6	101 ± 8	20.50 ± 3.79 (7.6)	88 ± 11	9.20 ± 1.62 (16.9)
UKF-NB-3^{r}CDDP^{1000}	5.32 ± 1.21	93 ± 18	13.85 ± 1.42 (0.4)	102 ± 6	18.55 ± 1.34 (0.3)
UKF-NB-3^{r}DOCE^{10}	469.60 ± 113.08	105 ± 10	32.60 ± 7.85 (14.4)	113 ± 9	0.50 ± 0.04 (939)
UKF-NB-3^{r}DOX^{20}	15,700.05 ± 1,019	92 ± 7	34.40 ± 11.73 (456)	107 ± 15	1.70 ± 0.49 (9235)
UKF-NB-3^{r}ETO^{200}	7.97 ± 0.13	98 ± 17	4.40 ± 0.27 (1.8)	101 ± 14	5.00 ± 0.14 (1.6)
UKF-NB-3^{r}GEMCI^{10}	0.40 ± 0.01	91 ± 12	0.50 ± 0.04 (0.8)	104 ± 18	0.50 ± 0.06 (0.8)
UKF-NB-3^{r}OXALI^{4000}	1.80 ± 0.78	95 ± 9	0.97 ± 0.09 (1.9)	105 ± 10	1.08 ± 0.14 (1.7)
UKF-NB-3^{r}TOPO^{20}	7.40 ± 0.71	103 ± 5	2.00 ± 0.05 (3.7)	109 ± 13	5.13 ± 0.84 (1.4)
UKF-NB-3^{r}VCR^{10}	26.59 ± 6.37	94 ± 5	1.95 ± 0.28 (13.6)	115 ± 14	1.35 ± 0.21 (19.7)
UKF-NB-6	*0.65 ± 0.09*	*101 ± 4*	*0.58 ± 0.07 (1.1)*	*107 ± 6*	*0.57 ± 0.07 (1.1)*
UKF-NB-6^{r}CARBO^{2000}	16.83 ± 1.62	97 ± 13	13.31 ± 2.79 (1.3)	103 ± 12	7.27 ± 2.17 (2.3)
UKF-NB-6^{r}CDDP^{2000}	79.93 ± 7.14	94 ± 11	21.72 ± 1.87 (3.7)	105 ± 2	13.30 ± 6.47 (6.0)
UKF-NB-6^{r}DOCE^{10}	14.33 ± 4.08	92 ± 14	5.48 ± 1.01 (2.6)	101 ± 18	1.83 ± 0.03 (7.8)
UKF-NB-6^{r}DOX^{20}	11.80 ± 1.56	99 ± 16	1.35 ± 0.35 (8.7)	99 ± 14	0.60 ± 0.07 (19.7)
UKF-NB-6^{r}ETO^{200}	3.60 ± 0.01	101 ± 19	0.50 ± 0.04 (7.2)	104 ± 7	1.24 ± 0.47 (2.9)
UKF-NB-6^{r}GEMCI^{10}	2.10 ± 0.84	110 ± 3	1.54 ± 0.10 (1.4)	106 ± 9	1.36 ± 0.61 (1.5)
UKF-NB-6^{r}OXALI^{4000}	5.34 ± 0.71	96 ± 10	3.47 ± 0.07 (1.5)	89 ± 7	3.04 ± 0.73 (1.8)
UKF-NB-6^{r}TOPO^{20}	3.47 ± 0.81	91 ± 4	1.00 ± 0.08 (3.5)	107 ± 10	1.35 ± 0.78 (2.6)

UKF-NB-6^{r}VCR^{10}	49.30 ± 2.24	104 ± 5	3.60 ± 0.12 (13.7)	103 ± 16	0.70 ± 0.10 (70.4)
UKF-NB-6^{r}VINOR^{40}	228.52 ± 41.54	95 ± 8	8.63 ± 1.01 (26.5)	100 ± 19	0.74 ± 0.06 (309)

[1] effect of verapamil (5µM) or zosuquidar (1.25µM) alone on cell viability presented as percentage (mean ± S.D.) relative to untreated control

[2] fold sensitisation (YM155 IC_{50}/ YM155 IC_{50} in the presence of ABCB1 inhibitor)
