# Supplementary material for: Testing of the Survivin Suppressant YM155 in a Large Panel of Drug-Resistant Neuroblastoma Cell Lines
Source: Cancers (Basel). 2020 Mar 2;12(3):577. doi: 10.3390/cancers12030577 (PMC7139505; doi:10.3390/cancers12030577)
Supplement: Supplementary file 1 [file cancers-12-00577-s001.zip › Michaelis et al_Supplements/Michaelis et al_Table S4_revised_02.pdf]

**Table S4**. YM155 concentrations that reduce the viability of neuroblastoma cell lines with varying p53 status by 50% ($IC_{50}$, mean ± S.D., n = 3) as indicated by MTT assay after 120h of incubation.

| **Cell line** | **p53 status** | **YM155 $IC_{50}$ (nM)** |
|---|---|---|
| UKF-NB-3 | wild-type | 0.49 ± 0.10 |
| UKF-NB-3$^{r}$Nutlin$^{10\mu M}$ | G245C (homo)[1] | 1.18 ± 0.07 (2.4)[2] |
| UKF-NB-3clone1 | wild-type | 0.35 ± 0.07 |
| UKF-NB-3clone1$^{r}$Nutlin$^{10\mu M}$I | stop codon in exon 4 | 0.40 ± 0.12 (1.1) |
| UKF-NB-3clone1$^{r}$Nutlin$^{10\mu M}$III | R248W (het) | 0.60 ± 0.08 (1.7) |
| UKF-NB-3clone1$^{r}$Nutlin$^{10\mu M}$IV | V173L (het) | 0.45 ± 0.06 (1.3) |
| UKF-NB-3clone1$^{r}$Nutlin$^{10\mu M}$VI | R196Q (het) | 0.55 ± 0.17 (1.6) |
| UKF-NB-3clone1$^{r}$Nutlin$^{10\mu M}$VIII | Y236C (het) | 0.50 ± 0.14 (1.4) |
| UKF-NB-3clone1$^{r}$Nutlin$^{10\mu M}$X | P151R (het) | 0.73 ± 0.08 (2.1) |
| UKF-NB-3clone3 | wild-type | 0.45 ± 0.06 |
| UKF-NB-3clone3$^{r}$Nutlin$^{10\mu M}$I | P152L (het) | 1.50 ± 0.06 (3.3) |
| UKF-NB-3clone3$^{r}$Nutlin$^{10\mu M}$VIII | N239S (het) | 0.50 ± 0.08 (1.1) |
| UKF-NB-3clone3$^{r}$Nutlin$^{10\mu M}$IX | R280S (het) | 1.03 ± 0.03 (2.3) |
| UKF-NB-3clone3$^{r}$Nutlin$^{10\mu M}$X | I251F (het) | 0.58 ± 0.09 (1.3) |
| UKF-NB-6 | wild-type | 0.65 ± 0.09 |
| UKF-NB-6$^{r}$Nutlin$^{10\mu M}$ | K132N (het); P223L (hom) | 0.64 ± 0.04 (1.0) |
| UKF-NB-6$^{r}$Nutlin$^{10\mu M}$I | S241F (hom) | 0.57 ± 0.01 (0.9) |
| UKF-NB-6$^{r}$Nutlin$^{10\mu M}$IV | C135F (het); D281Y (het) | 0.43 ± 0.04 (0.7) |

[1] homo = homozygous, het = heterozygous

[2] fold change YM155 $IC_{50}$ nutlin-3-resistant sub-line/ YM155 $IC_{50}$ respective parental cell line
