# Supplementary material for: Testing of the Survivin Suppressant YM155 in a Large Panel of Drug-Resistant Neuroblastoma Cell Lines
Source: Cancers (Basel). 2020 Mar 2;12(3):577. doi: 10.3390/cancers12030577 (PMC7139505; doi:10.3390/cancers12030577)
Supplement: Supplementary file 1 [file cancers-12-00577-s001.zip › Michaelis et al_Supplements/Michaelis et al_Figure 6_revised.pptx]

## Slide 1
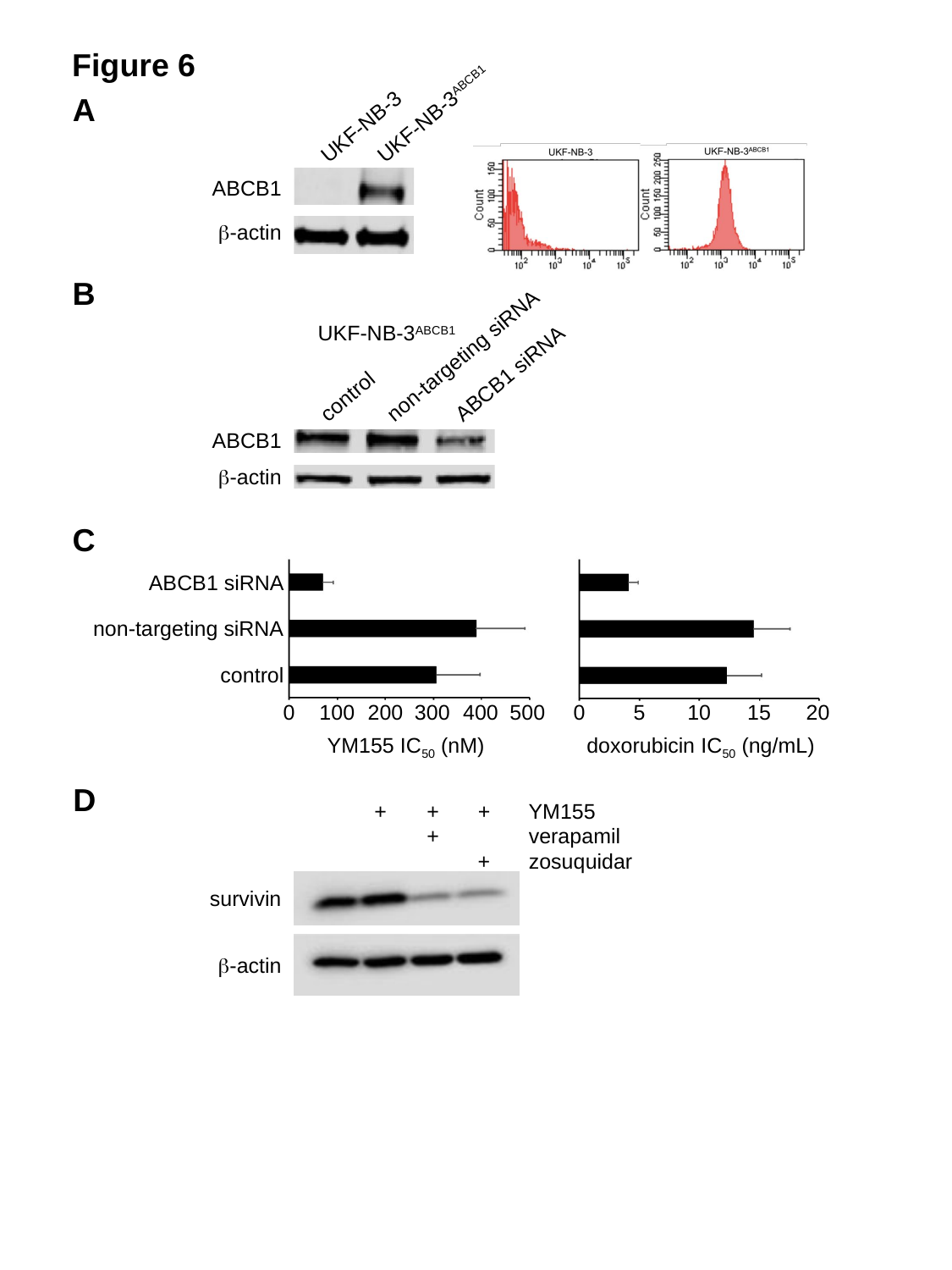

Figure 6
A
UKF-NB-3ABCB1
UKF-NB-3
ABCB1
β-actin
B
UKF-NB-3ABCB1
non-targeting siRNA
ABCB1 siRNA
control
ABCB1
β-actin
C
ABCB1 siRNA
non-targeting siRNA
control
0
100
200
300
400
500
0
5
10
15
20
YM155 IC50 (nM)
doxorubicin IC50 (ng/mL)
D
+
+
+
YM155
+
verapamil
+
zosuquidar
survivin
β-actin
