# Supplementary material for: Testing of the Survivin Suppressant YM155 in a Large Panel of Drug-Resistant Neuroblastoma Cell Lines
Source: Cancers (Basel). 2020 Mar 2;12(3):577. doi: 10.3390/cancers12030577 (PMC7139505; doi:10.3390/cancers12030577)
Supplement: Supplementary file 1 [file cancers-12-00577-s001.zip › Michaelis et al_Supplements/Michaelis et al_Figure 1_revised.pptx]

## Slide 1
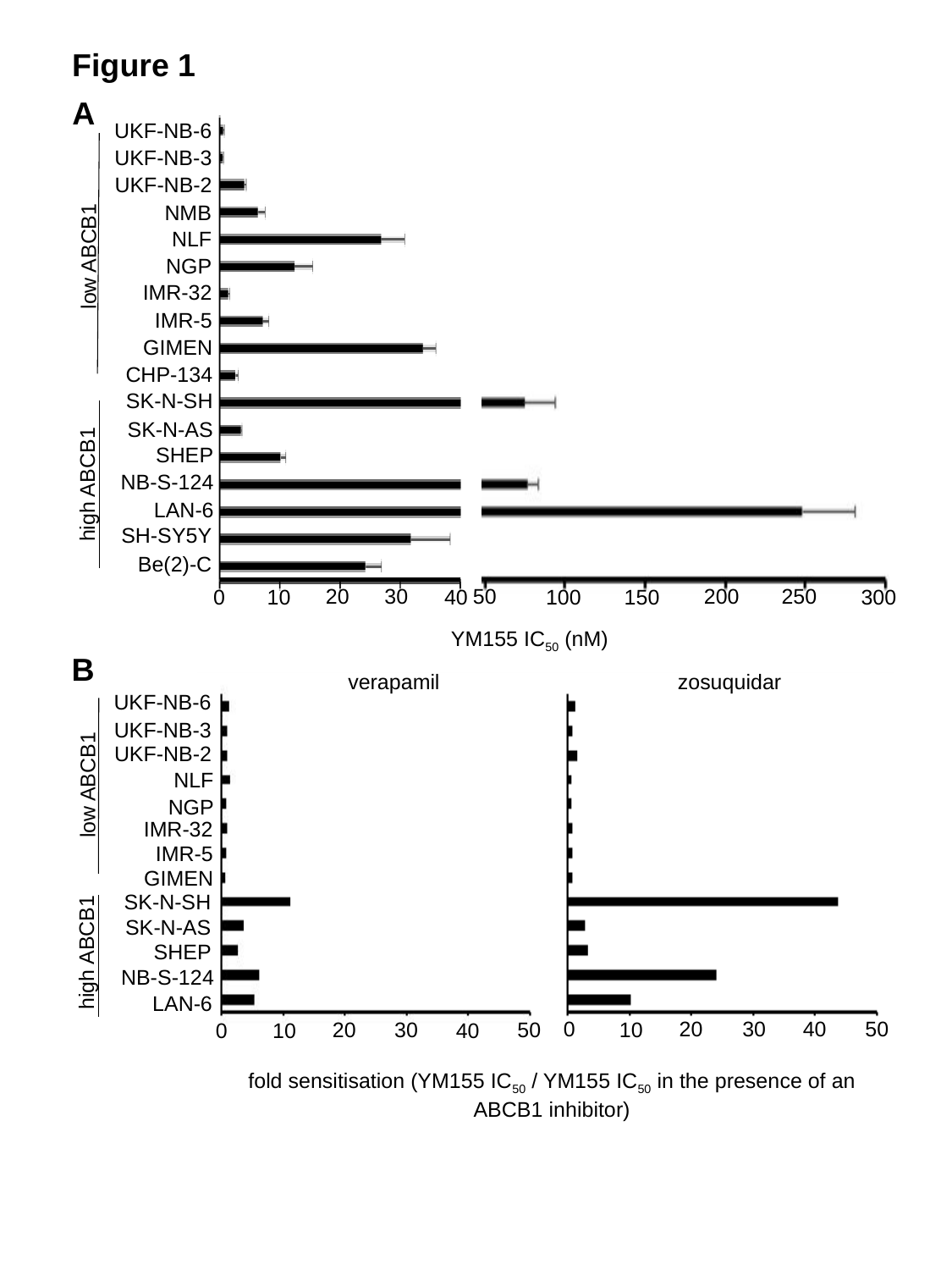

Figure 1
A
UKF-NB-6
UKF-NB-3
UKF-NB-2
NMB
NLF
low ABCB1
NGP
IMR-32
IMR-5
GIMEN
CHP-134
SK-N-SH
SK-N-AS
SHEP
NB-S-124
high ABCB1
LAN-6
SH-SY5Y
Be(2)-C
50
20
200
30
250
40
300
0
100
10
150
YM155 IC50 (nM)
B
verapamil
zosuquidar
UKF-NB-6
UKF-NB-3
UKF-NB-2
NLF
low ABCB1
NGP
IMR-32
IMR-5
GIMEN
SK-N-SH
SK-N-AS
SHEP
high ABCB1
NB-S-124
LAN-6
30
20
40
50
0
20
10
30
50
0
40
10
fold sensitisation (YM155 IC50 / YM155 IC50 in the presence of an ABCB1 inhibitor)
