# Supplementary material for: Testing of the Survivin Suppressant YM155 in a Large Panel of Drug-Resistant Neuroblastoma Cell Lines
Source: Cancers (Basel). 2020 Mar 2;12(3):577. doi: 10.3390/cancers12030577 (PMC7139505; doi:10.3390/cancers12030577)
Supplement: Supplementary file 1 [file cancers-12-00577-s001.zip › Michaelis et al_Supplements/Michaelis et al_Table S3_revised_02.pdf]

Table S3. YM155 concentrations that reduce the viability of MYCN-amplified and non-MYCN-amplified neuroblastoma cell lines by 50% (IC_{50}, mean ± S.D., n = 3) in the absence or presence of the ABCB1 inhibitors verapamil (5 µM) or zosuquidar (1.25 µM) as indicated by MTT assay after 120h of incubation.

**Cell line**	**YM155 IC_{50} (nM)**	**+ verapamil (5 µM)[1] YM155 IC_{50} (nM)**	**+ zosuquidar (1.25 µM) YM155 IC_{50} (nM)**
MYCN amplification[2]			
CHP-134 (wt)[3]	2.64 ± 0.50	1.64 ± 0.27	1.85 ± 0.34
IMR-5 (wt)	7.18 ± 1.04	9.70 ± 1.97	10.64 ± 2.80
IMR-32 (wt)	1.40 ± 0.35	1.70 ± 0.41	1.80 ± 0.23
NB-S-124 (wt)	76.66 ± 6.51	12.52 ± 1.16	3.20 ± 0.40
NGP (wt)	12.48 ± 3.01	17.35 ± 4.97	24.95 ± 0.21
NLF (V203M)	26.78 ± 4.04	19.55 ± 1.20	45.30 ± 1.34
UKF-NB-2 (wt)	4.18 ± 0.27	4.55 ± 0.32	2.85 ± 0.14
UKF-NB-3 (wt)	0.49 ± 0.10	0.61 ± 0.13	0.74 ± 0.10
UKF-NB-6 (wt)	0.65 ± 0.09	0.58 ± 0.07	0.57 ± 0.07
no MYCN amplification			
GIMEN (wt)	33.74 ± 2.26	52.90 ± 8.62	50.87 ± 5.91
LAN-6 (wt)	248.1 ± 32.9	46.75 ± 2.33	24.35 ± 1.06
SHEP (wt)	10.15 ± 0.84	3.92 ± 0.11	3.20 ± 0.14
SK-N-AS (null)	3.55 ± 0.21	1.01 ± 0.26	1.31 ± 0.11
SK-N-SH (wt)	74.94 ± 19.52	6.80 ± 0.83	1.72 ± 0.15

[1] Effects of verapamil or zosuquidar alone on cell viability are presented in Table S5.

[2] References:

Thiele, C. Neuroblastoma. *In Human Cell Culture*, Masters, J., Ed., Kluwer Academic Publishers: Lancaster, UK, 1998; Volume 1, pp. 21-53.

Fischer, M.; Berthold, F. Characterization of the gene expression profile of neuroblastoma cell line IMR-5 using serial analysis of gene expression. *Cancer Lett.* **2003**, *190*, 79-87.

Kotchetkov, R.; Cinatl, J.; Blaheta, R.; Vogel, J.U.; Karaskova, J.; Squire, J.; Hernáiz Driever, P.; Klingebiel, T.; Cinatl, J. Jr. Development of resistance to vincristine and doxorubicin in neuroblastoma alters malignant properties and induces additional karyotype changes: a preclinical model. *Int. J. Cancer* **2003**, *104*, 36-43.
Edsjö, A.; Nilsson, H.; Vandesompele, J.; Karlsson, J.; Pattyn, F.; Culp, L.A.; Speleman, F.; Påhlman, S. Neuroblastoma cells with overexpressed MYCN retain their capacity to undergo neuronal differentiation. *Lab. Invest.* **2004**, *84*, 406-417.
Kotchetkov, R.; Driever, P.H.; Cinatl, J.; Michaelis, M.; Karaskova, J.; Blaheta, R.; Squire, J.A.; Von Deimling, A.; Moog, J.; Cinatl, J. Jr. Increased malignant behavior in neuroblastoma cells with acquired multi-drug resistance does not depend on P-gp expression. *Int. J. Oncol.* **2005**, *27*, 1029-1037.
Carr, J.; Bown, N.P.; Case, M.C.; Hall, A.G.; Lunec, J.; Tweddle, D.A. High-resolution analysis of allelic imbalance in neuroblastoma cell lines by single nucleotide polymorphism arrays. *Cancer Genet. Cytogenet.* **2007**, *172*, 127-138.
Michaelis, M.; Kleinschmidt, M.C.; Barth, S.; Rothweiler, F.; Geiler, J.; Breitling, R.; Mayer, B.; Deubzer, H.; Witt, O.; Kreuter, J.; Doerr, H.W.; Cinatl, J.; Cinatl, J. Jr. Anti-cancer effects of artesunate in a panel of chemoresistant neuroblastoma cell lines. *Biochem. Pharmacol.* **2010**, *79*, 130-136.
Dassi, E.; Greco, V.; Sidarovich, V.; Zuccotti, P.; Arseni, N.; Scaruffi, P.; Tonini, G.P.; Quattrone, A. Multi-omic profiling of MYCN-amplified neuroblastoma cell-lines. *Genom. Data* **2015**, *6*, 285-287.
Michaelis, M.; Agha, B.; Rothweiler, F.; Löschmann, N.; Voges, Y.; Mittelbronn, M.; Starzetz, T.; Harter, P.N.; Abhari, B.A.; Fulda, S.; Westermann, F.; Riecken, K.; Spek, S.; Langer, K.; Wiese, M.; Dirks, W.G.; Zehner, R.; Cinatl, J.; Wass, M.N.; Cinatl, J. Jr. Identification of flubendazole as potential anti-neuroblastoma compound in a large cell line screen. *Sci. Rep.* **2015**, *5*, 8202.
[3] *TP53* status: wt, wild-type; otherwise type of mutation is provided
