# Supplementary material for: Testing of the Survivin Suppressant YM155 in a Large Panel of Drug-Resistant Neuroblastoma Cell Lines
Source: Cancers (Basel). 2020 Mar 2;12(3):577. doi: 10.3390/cancers12030577 (PMC7139505; doi:10.3390/cancers12030577)
Supplement: Supplementary file 1 [file cancers-12-00577-s001.zip › Michaelis et al_Supplements/Michaelis et al_Table S2_revised_02.pdf]

Table S2. ABCB1 status of neuroblastoma cell lines.

Low ABCB1

UKF-NB-6, described in:

Löschmann, N.; Michaelis, M.; Rothweiler, F.; Voges, Y.; Balónová, B.; Blight, B.A.; Cinatl, J Jr. ABCB1 as predominant resistance mechanism in cells with acquired SNS-032 resistance. *Oncotarget* **2016**, *7*, 58051-58064. doi: 10.18632/oncotarget.11160.

UKF-NB-3, described in:

Kotchetkov, R.; Driever, P.H.; Cinatl, J.; Michaelis, M.; Karaskova, J.; Blaheta, R.; Squire, J.A.; Von Deimling, A.; Moog, J.; Cinatl, J. Jr. Increased malignant behavior in neuroblastoma cells with acquired multi-drug resistance does not depend on P-gp expression. *Int. J. Oncol.* **2005**, *27*, 1029-1037.

UKF-NB-2, described in:

Kotchetkov, R.; Cinatl, J.; Blaheta, R.; Vogel, J.U.; Karaskova, J.; Squire, J.; Hernáiz Driever, P.; Klingebiel, T.; Cinatl, J. Jr. Development of resistance to vincristine and doxorubicin in neuroblastoma alters malignant properties and induces additional karyotype changes: a preclinical model. *Int. J. Cancer* **2003**, *104*, 36-43. doi: 10.1002/ijc.10917

NMB, described in:

Kurowski, C.; Berthold, F. Presence of classical multidrug resistance and P-glycoprotein expression in human neuroblastoma cells. *Ann. Oncol*. **1998**, 9, 1009-1014. doi: 10.1023/A:1008476528846

NLF, described in

Löschmann, N.; Michaelis, M.; Rothweiler, F.; Voges, Y.; Balónová, B.; Blight, B.A.; Cinatl, J Jr. ABCB1 as predominant resistance mechanism in cells with acquired SNS-032 resistance. *Oncotarget* **2016**, *7*, 58051-58064. doi: 10.18632/oncotarget.11160.

NGP:

NGP cells do not become sensitised to the ABCB1 substrate vincristine by the ABCB1 inhibitors verapamil or zosuquidar, which indicates together with the YM155 sensitivity data (Table S6) that these cells do not display ABCB1-mediated drug resistance:

		+ verapamil (5μM)		+ zosuquidar (1.25μM)	
	IC_{50} vincristine	verapamil alone	IC_{50} vincristine	zosuquidar alone	IC_{50} vincristine
NGP	4.38 ± 0.87	102 ± 14[1]	3.93 ± 0.69	97 ± 16	5.24 ± 0.72

[1] effect of verapamil (5μM) or zosuquidar (1.25μM) alone on cell viability presented as percentage (mean ± S.D.) relative to untreated control

IMR-32, described in:

Kurowski, C.; Berthold, F. Presence of classical multidrug resistance and P-glycoprotein expression in human neuroblastoma cells. *Ann. Oncol*. **1998**, 9, 1009-1014. doi: 10.1023/A:1008476528846

IMR-5, described in:

Löschmann, N.; Michaelis, M.; Rothweiler, F.; Zehner, R.; Cinatl, J.; Voges, Y.; Sharifi, M.; Riecken, K.; Meyer, J.; von Deimling, A.; Fichtner, I.; Ghafourian, T.; Westermann, F.; Cinatl, J Jr. Testing of SNS-032 in a Panel of Human Neuroblastoma Cell Lines with Acquired Resistance to a Broad Range of Drugs. *Transl. Oncol.* **2013**, *6*, 685-696. doi: 10.1593/tlo.13544

GIMEN, described in:
Kurowski, C.; Berthold, F. Presence of classical multidrug resistance and P-glycoprotein expression in human neuroblastoma cells. *Ann. Oncol.* **1998**, 9, 1009-1014. doi: 10.1023/A:1008476528846

CHP-134, described in:
Kurowski, C.; Berthold, F. Presence of classical multidrug resistance and P-glycoprotein expression in human neuroblastoma cells. *Ann. Oncol.* **1998**, 9, 1009-1014. doi: 10.1023/A:1008476528846

High ABCB1
SK-N-SH, described in:
Lamers, F.; Schild, L.; Koster, J.; Versteeg, R.; Caron, H.N.; Molenaar, J.J. Targeted BIRC5 silencing using YM155 causes cell death in neuroblastoma cells with low ABCB1 expression. *Eur. J. Cancer* **2012**, *48*, 763-771. doi: 10.1016/j.ejca.2011.10.012

SK-N-AS, described in:
Dijkhuis, A.J.; Douwes, J.; Kamps, W.; Sietsma, H.; Kok, J.W. Differential expression of sphingolipids in P-glycoprotein or multidrug resistance-related protein 1 expressing human neuroblastoma cell lines. *FEBS Lett.* **2003**, *548*, 28-32. doi: 10.1016/s0014-5793(03)00721-x

SHEP, described in:
Löschmann, N.; Michaelis, M.; Rothweiler, F.; Voges, Y.; Balónová, B.; Blight, B.A.; Cinatl, J Jr. ABCB1 as predominant resistance mechanism in cells with acquired SNS-032 resistance. *Oncotarget* **2016**, *7*, 58051-58064. doi: 10.18632/oncotarget.11160.

LAN-6:
LAN-6 cells become sensitised to the ABCB1 substrate vincristine by the ABCB1 inhibitors verapamil or zosuquidar, which indicates together with the YM155 sensitivity data (Table S6) that these cells display an ABCB1-mediated drug resistance phenotype:

		+ verapamil (5µM)		+ zosuquidar (1.25µM)	
	IC_{50} vincristine	verapamil alone	IC_{50} vincristine	zosuquidar alone	IC_{50} vincristine
LAN-6	92.5 ± 14.2	88 ± 10[1]	16.8 ± 4.9	105 ± 6	8.3 ± 2.6

[1] effect of verapamil (5µM) or zosuquidar (1.25µM) alone on cell viability presented as percentage (mean ± S.D.) relative to untreated control

NB-S-124:
NB-S-124 cells become sensitised to the ABCB1 substrate vincristine by the ABCB1 inhibitors verapamil or zosuquidar, which indicates together with the YM155 sensitivity

data (Table S6) that these cells display an ABCB1-mediated drug resistance phenotype:

		+ verapamil (5µM)		+ zosuquidar (1.25µM)	
	IC_{50} vincristine	verapamil alone	IC_{50} vincristine	zosuquidar alone	IC_{50} vincristine
NB-S-124	69.2 ± 18.3	99 ± 17[1]	13.7 ± 3.8	108 ± 7	14.6 ± 4.2

[1] effect of verapamil (5µM) or zosuquidar (1.25µM) alone on cell viability presented as percentage (mean ± S.D.) relative to untreated control

SH-SY5Y, described in:
Lamers, F.; Schild, L.; Koster, J.; Versteeg, R.; Caron, H.N.; Molenaar, J.J. Targeted BIRC5 silencing using YM155 causes cell death in neuroblastoma cells with low ABCB1 expression. *Eur. J. Cancer* **2012**, *48*, 763-771. doi: 10.1016/j.ejca.2011.10.012

Be(2)C, described in:
LaQuaglia, M.P.; Kopp, E.B.; Spengler, B.A.; Meyers, M.B.; Biedler, J.L. Multidrug resistance in human neuroblastoma cells. *J. Pediatr. Surg.* **1991**, *26*, 1107-1112. doi: 10.1016/0022-3468(91)90684-I
