# Supplementary material for: Testing of the Survivin Suppressant YM155 in a Large Panel of Drug-Resistant Neuroblastoma Cell Lines
Source: Cancers (Basel). 2020 Mar 2;12(3):577. doi: 10.3390/cancers12030577 (PMC7139505; doi:10.3390/cancers12030577)
Supplement: Supplementary file 1 [file cancers-12-00577-s001.zip › Michaelis et al_Supplements/Michaelis et al_Figure 7_revised.pptx]

## Slide 1
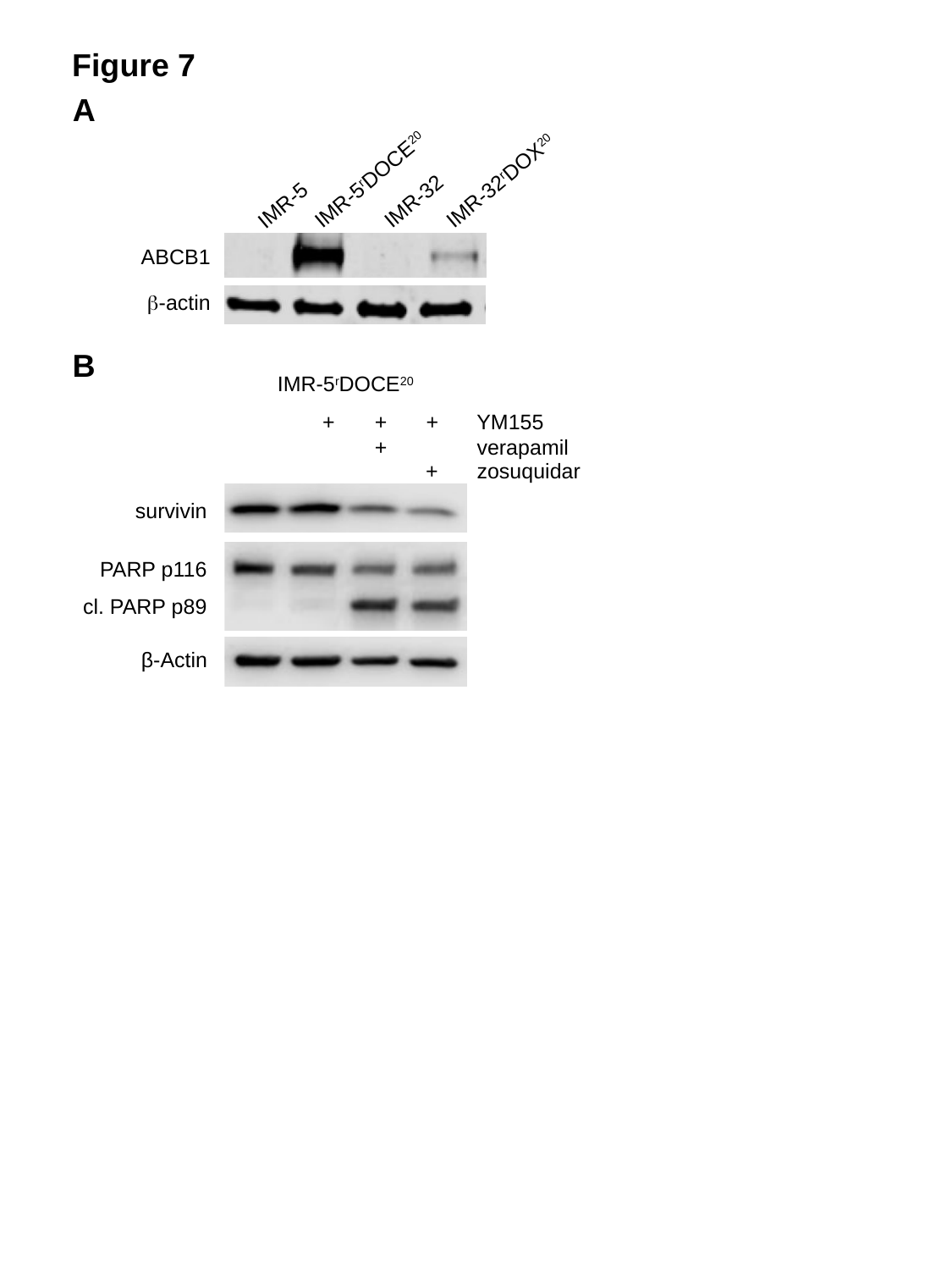

Figure 7
A
IMR-32
IMR-32rDOX20
IMR-5rDOCE20
IMR-5
ABCB1
β-actin
B
IMR-5rDOCE20
+
+
+
YM155
+
verapamil
+
zosuquidar
survivin
PARP p116
cl. PARP p89
β-Actin
