# Supplementary material for: Testing of the Survivin Suppressant YM155 in a Large Panel of Drug-Resistant Neuroblastoma Cell Lines
Source: Cancers (Basel). 2020 Mar 2;12(3):577. doi: 10.3390/cancers12030577 (PMC7139505; doi:10.3390/cancers12030577)
Supplement: Supplementary file 1 [file cancers-12-00577-s001.zip › Michaelis et al_Supplements/Michaelis et al_Table S5_revised_02.pdf]

Table S5. Mean YM155 concentrations that reduce the viability of neuroblastoma cell lines with resistance to certain drug classes by 50% (IC_{50}, mean ± S.D., n = 3) as indicated by MTT assay after 120h of incubation. Values are presented as mean ± S.D. Individual values are presented in Table S1.

Drug class	YM155 IC_{50} (nM)
topoisomerase I inhibitors	4.63 ± 2.52
parental	7.91 ± 8.27
nucleoside analogue (gemcitabine)	9.18 ± 14.62
alkylating agents	18.29 ± 5.63
platinum drugs	58.43 ± 98.80
topoisomerase II inhibitors	73.67 ± 112.42
including UKF-NB-3^{r}DOX[20]	1190 ± 4026
taxane (docetaxel)	354 ± 411
including IMR-5^{r}DOCE[20]	3889 ± 7908
vinca alkaloids	1725 ± 2519
