# Supplementary material for: Testing of the Survivin Suppressant YM155 in a Large Panel of Drug-Resistant Neuroblastoma Cell Lines
Source: Cancers (Basel). 2020 Mar 2;12(3):577. doi: 10.3390/cancers12030577 (PMC7139505; doi:10.3390/cancers12030577)
Supplement: Supplementary file 1 [file cancers-12-00577-s001.zip › Michaelis et al_Supplements/Michaelis et al_Figure S3_revised.pdf]

Figure S3

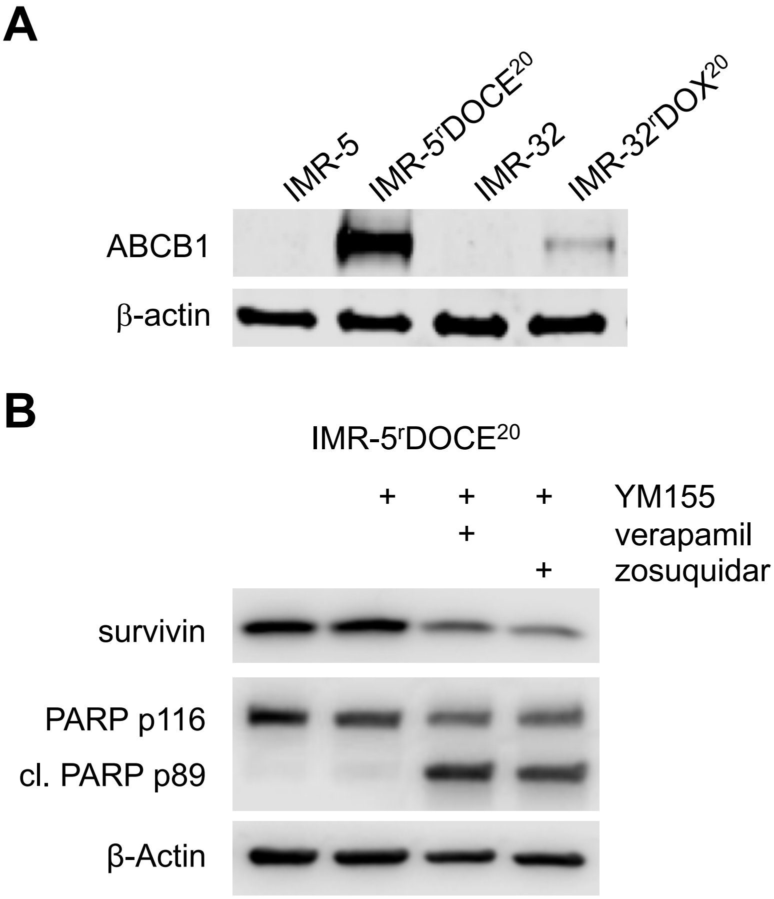

Figure S3. ABCB1 expression and YM155 activity in drug-adapted neuroblastoma cells. A) Representative Western blots indicating ABCB1 levels in IMR-5, IMR-5^{r}DOCE^{20}, IMR-32, and IMR-32^{r}DOX^{20}. B) Effects of YM155 (500nM) on survivin levels and PARP cleavage in IMR-5^{r}DOCE^{20} cells in the presence or absence of verapamil (5μM) or zosuquidar (1.25μM) after 24h of incubation.

Figure S3

Uncropped blots

A

ABCB1

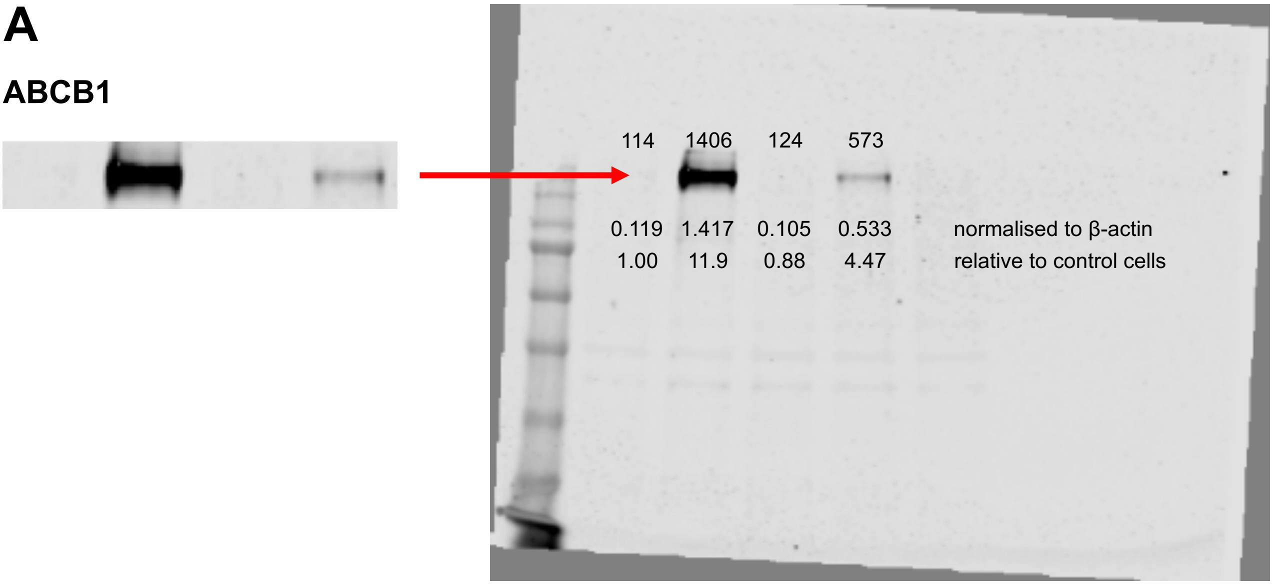

β-actin

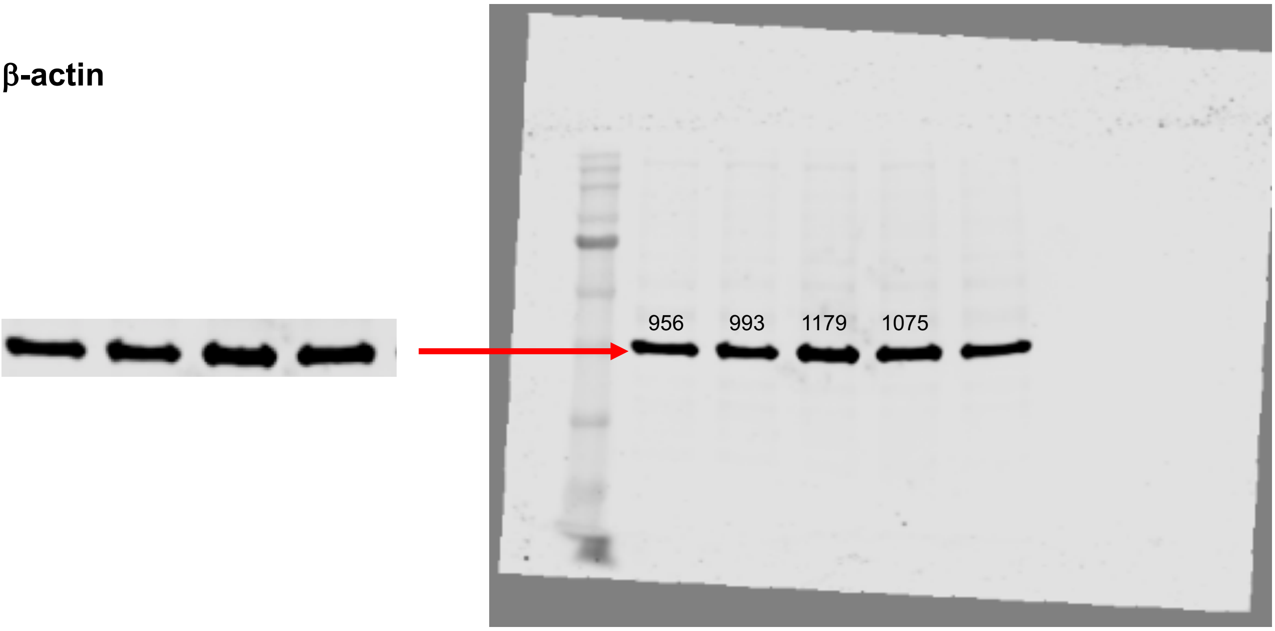

Densitometric analysis was performed with QuantiOne (BioRad). ABCB1 levels were normalised to β-actin expression and values relative to control cells are displayed.

Figure S3
Uncropped blots
B

survivin

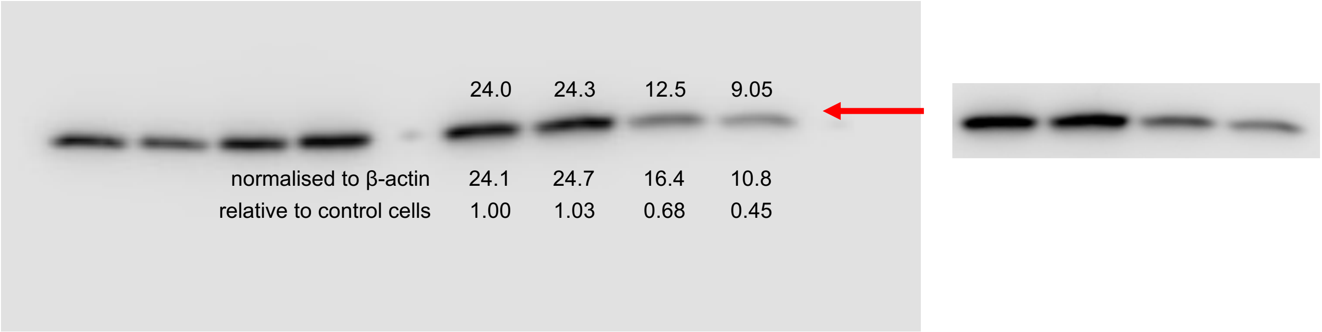

PARP

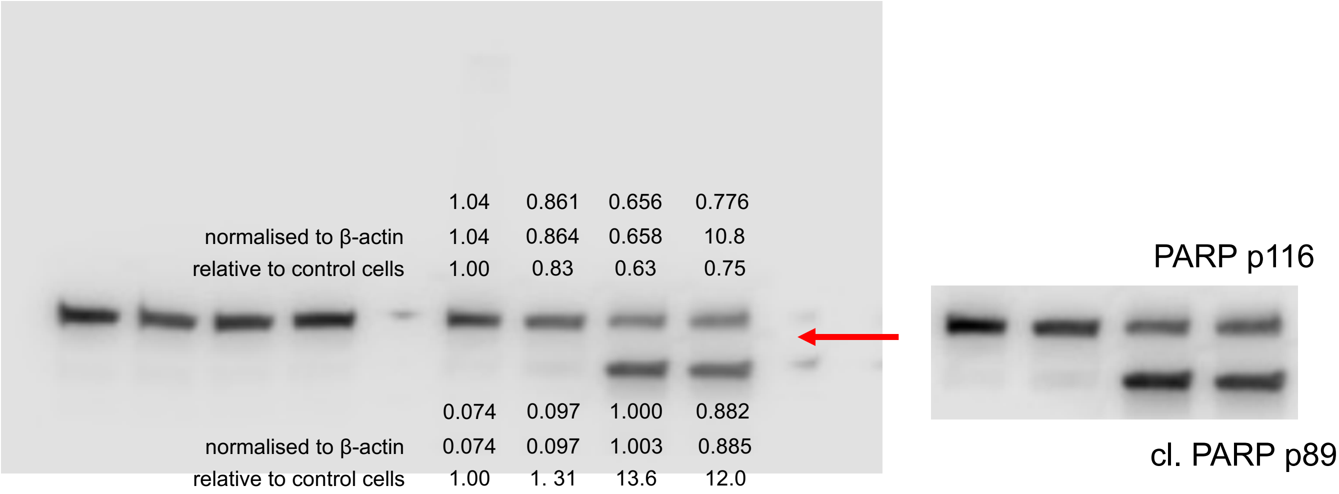

β-actin

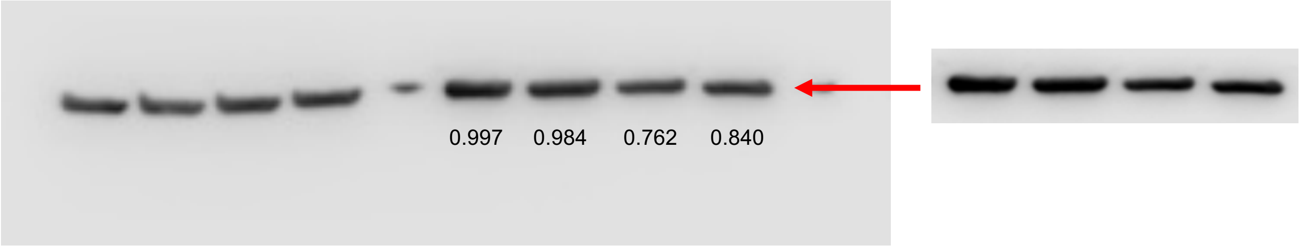

Densitometric analysis was performed with Image Studio Ver. 5.2 software (LICOR). Survivin and PARP levels were normalised to β-actin expression and values relative to control cells are displayed.
