# Supplementary figures and images for: Testing of the Survivin Suppressant YM155 in a Large Panel of Drug-Resistant Neuroblastoma Cell Lines
Source: Cancers (Basel). 2020 Mar 2;12(3):577. doi: 10.3390/cancers12030577 (PMC7139505; doi:10.3390/cancers12030577)

## Slide 1
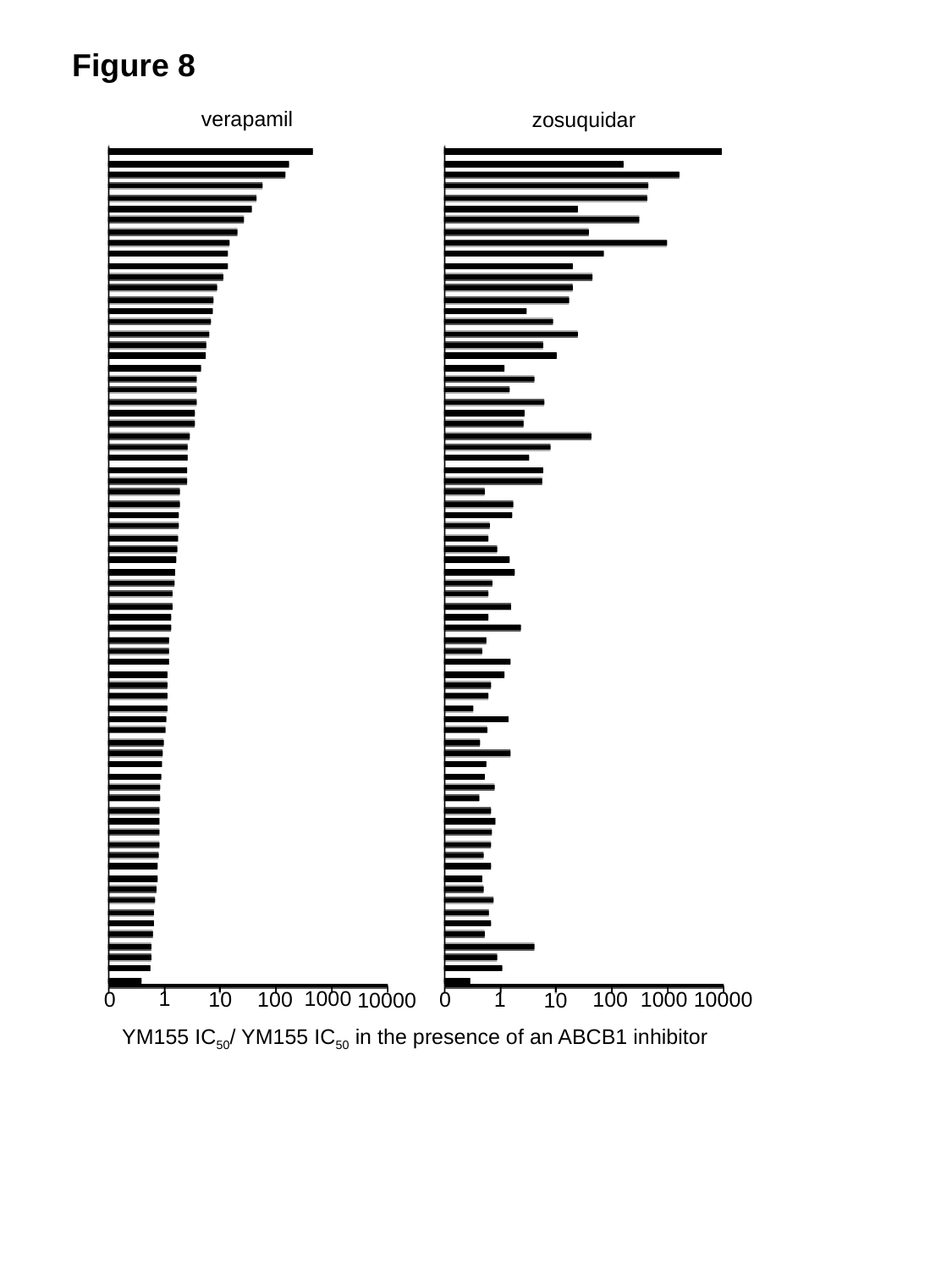

Figure 8
verapamil
zosuquidar
1
1000
0
1
100
1000
0
10
100
10000
10
10000
YM155 IC50/ YM155 IC50 in the presence of an ABCB1 inhibitor

Supplement: Supplementary file 1 [file cancers-12-00577-s001.zip › Michaelis et al_Supplements/Michaelis et al_Figure 8_revised.pptx]
